# Friend or Foe? Impacts of Dietary Xylans, Xylooligosaccharides, and Xylanases on Intestinal Health and Growth Performance of Monogastric Animals

**DOI:** 10.3390/ani11030609

**Published:** 2021-02-26

**Authors:** Jonathan T. Baker, Marcos E. Duarte, Debora M. Holanda, Sung Woo Kim

**Affiliations:** Department of Animal Science, North Carolina State University, Raleigh, NC 27695, USA; jtbaker3@ncsu.edu (J.T.B.); mduarte@ncsu.edu (M.E.D.); dmurato@ncsu.edu (D.M.H.)

**Keywords:** intestinal health, growth performance, poultry, prebiotics, swine, xylooligosaccharides

## Abstract

**Simple Summary:**

Xylan is naturally present in typical feedstuffs fed to animals and has been shown to cause increased digesta viscosity reducing nutrient digestibility and growth. Xylooligosaccharides are sugar oligomers consisting of xylose units that can be extracted and purified from biomaterials for use as a prebiotic in monogastric feeds. Xylooligosaccharides can also be obtained from the hydrolysis of xylan either in the intestine of the animal or in-vitro through various techniques. The question of xylanase supplementation versus xylooligosaccharide supplementation as well as symbiosis of both on the intestinal health and performance of monogastric livestock is still up for debate. Xylanase inhibitors present in common cereal grains provide yet another obstacle to overcome and are found to be highly variable. As the fear of antibiotic resistance increases, novel approaches to improve growth performance and enhance intestinal health without the use of antibiotics also increase. The aim of this article is to review the structural difference and its impact on xylan in feeds, classification and the use of various xylanases, as well as the production and use of xylooligosaccharides for the physiological effects on intestinal health and growth performance of monogastric animals.

**Abstract:**

This paper discusses the structural difference and role of xylan, procedures involved in the production of xylooligosaccharides (XOS), and their implementation into animal feeds. Xylan is non-starch polysaccharides that share a β-(1-4)-linked xylopyranose backbone as a common feature. Due to the myriad of residues that can be substituted on the polymers within the xylan family, more anti-nutritional factors are associated with certain types of xylan than others. XOS are sugar oligomers extracted from xylan-containing lignocellulosic materials, such as crop residues, wood, and herbaceous biomass, that possess prebiotic effects. XOS can also be produced in the intestine of monogastric animals to some extent when exogenous enzymes, such as xylanase, are added to the feed. Xylanase supplementation is a common practice within both swine and poultry production to reduce intestinal viscosity and improve digestive utilization of nutrients. The efficacy of xylanase supplementation varies widely due a number of factors, one of which being the presence of xylanase inhibitors present in common feedstuffs. The use of prebiotics in animal feeding is gaining popularity as producers look to accelerate growth rate, enhance intestinal health, and improve other production parameters in an attempt to provide a safe and sustainable food product. Available research on the impact of xylan, XOS, as well as xylanase on the growth and health of swine and poultry, is also summarized. The response to xylanase supplementation in swine and poultry feeds is highly variable and whether the benefits are a result of nutrient release from NSP, reduction in digesta viscosity, production of short chain xylooligosaccharides or a combination of these is still in question. XOS supplementation seems to benefit both swine and poultry at various stages of production, as well as varying levels of XOS purity and degree of polymerization; however, further research is needed to elucidate the ideal dosage, purity, and degree of polymerization needed to confer benefits on intestinal health and performance in each respective species.

## 1. Introduction

Following cellulose, xylan is the second most abundant renewable polysaccharide in nature [1]. Monogastric animals such as poultry and swine do not endogenously synthesize the necessary enzymes for hydrolysis of non-starch polysaccharides (NSPs) such as xylan [2]. The absence of endogenous enzymes to degrade xylan allows the NSPs to encapsulate other nutrients and act as a barrier for digesting those trapped nutrients in the small intestine, subsequently increasing the viscosity of digesta [3]. A decrease in growth performance caused by increased digesta viscosity is largely observed in poultry but also in swine consuming feeds containing a considerable amount of NSPs based on results from recent studies [4,5,6].

In response to this issue, livestock producers have implemented exogenous enzymes such as xylanases into the feeds fed to swine and poultry to degrade xylan to short chain sugars, thus reducing intestinal viscosity and improving the digestive utilization of nutrients [7,8]. Xylan from lignocellulosic biomass can also be hydrolyzed through exogenous chemical and enzymatic processes to produce xylooligosaccharide (XOS) mixtures that are classified as prebiotics [2]. Lignocellulosic biomass is the most economical and renewable natural resource in the world [9] and includes terrestrial plants, such as trees and grasses, as well as agricultural biomass waste, such as corn stover, straw, saw-mill waste, paper mill discards and energy crops.

The emergence of antibiotics for the treatment of clinical infections and sickness in livestock has had a significant and positive impact on the health and welfare of animals. Due to increased consumer awareness and fear of antimicrobial resistance, however, the use of low concentrations of antibiotics within animal feed as a “growth promotant” is quickly becoming obsolete within the United States. The European Union has banned the use of common antibiotic growth promotors such as Tylosin, Spiramycin, Bacitracin, and Virginiamycin for use in animal feeds since 2006. In recent years, the animal feed industry has placed emphasis on the investigation and use of eubiotics as a replacement for antibiotics as growth promotants [10]. These non-antibiotic growth promotants include essential oils, exogenous enzymes, herbs, organic acids, prebiotics and probiotics. Prebiotics are a class of molecules that possess the potential to substantially affect the physiology of the whole body, thus improving health and well-being [11]. Prebiotics are non-digestible feed components that may be fermented by beneficial bacteria in the intestine. During fermentation of prebiotics, production of volatile fatty acids (VFAs) such as acetic, propionic, and butyric acid typically increase thus lowering the pH within the intestine [3]. This is significant as pathogenic bacteria, such as Salmonella, Escherichia coli and Clostridium, have shown impaired proliferation in acidic environments [7]. In addition, the increase in production of butyric acid is associated with the proliferation and differentiation of epithelial cells in the gastrointestinal tract, thus expanding the surface area available for absorption of nutrients [7]. Prebiotics have been investigated as an alternative to antibiotic growth promoters as studies have shown preferential stimulation of growth in advantageous bacteria such as *Bifidobacterium* and other lactic acid bacteria in the gastrointestinal tract leading to an increase in the concentration of short chain fatty acids, which is often associated with a decrease in pathogenic bacteria [12,13]. Among prebiotics, XOS exhibits great potential to maintain and improve intestinal microbiota for enhanced health and growth in various animal species, specifically swine and poultry.

In pigs, weaning stress is characterized by negatively affecting the functions of the intestine that include digestion, absorption, secretion, and immunity [14,15,16,17]. Weaning affects the energy and protein metabolism as well as the cellular macromolecule organization which, then, influences the proliferation of intestinal epithelial cells in weaned piglets [17]. Thus, implementing products such as prebiotics that improve intestinal function has been of great interest to aid piglets in recovering from weaning stress and promotes growth performance. In broiler chickens, feed additives such as prebiotics and carbohydrases have seen wide use to improve intestinal health as well as stimulate performance [18]. This review is focused on the structural difference and role of xylan in lignocellulosic materials as well as the use of xylanases and the extraction of XOS and its implementation into swine and poultry feeds as a prebiotic to enhance intestinal health and growth performance. 

## 2. Structure, Occurrence and Characterization of Xylans and Their Derivatives

The plant cell wall is central to intercellular communication and plays an essential role in plant–microbe interactions. Typically, plant cell walls are divided into two categories of primary and secondary walls. The former is used to describe cell walls that surround cells capable of growth or those that are actively growing. Primary cell walls are composed of hemicellulosic polysaccharides, cellulose microfibrils, and pectic polysaccharides [19]. As time passes and the cell becomes specialized, different layers of polymers start being deposited, forming the secondary cell wall. Secondary cell walls are usually described as thickened structures that contain lignin and polysaccharides such as cellulose and hemicellulose [19]. Various hemicelluloses constitute the secondary cell wall such as xylan, heteroxylan, xyloglucan, arabinogalactans, and glucomannan [20]. 

Common cereal grains and related coproducts used in the feeding of livestock contain variable amounts of NSP. Non-starch polysaccharides have been shown to increase digesta viscosity within the small intestines, resulting in depressed absorption and digestibility of nutrients [21,22,23,24]. In addition, increased digesta viscosity has been linked to an increase in the pathogenic load within the small intestine which could result in an increased level of oxidative stress as well as inflammatory responses in the small intestine of nursery pigs [23,24]. Viscosity is related to physical properties of the polysaccharide such as structure and molecular weight rather than the type of linkage or sugar composition of the polysaccharide [25]. Relative NSP (%) with soluble and insoluble components in common feedstuffs can be seen in Table 1. 

Between 10 and 30% of the composition of cereals is NSP with only trace amounts containing pectic polymers and the bulk consisting of arabinoxylan, β-glucans, and cellulose [25]. Common cereal grains used in animal feeding can be grouped into two classifications, viscous and non-viscous cereals, based on the concentration of soluble NSP present within the respective grain [25]. Cereals included within the viscous classification are barley, oats, triticale, and wheat, while common cereals in the non-viscous group include corn, millet, rice and sorghum [25]. The arabinoxylan and β-glucans present in the viscous cereal grain group are partially soluble and have been shown to form more viscous solutions when digested, hence the distinction in the two classifications. The extent of solubility of NSPs typically refers to its solubility in water and has a significant impact on the nutritional properties of NSP when fed to monogastric animals [25]. Cereal by-products usually contain higher amounts of insoluble NSP and therefore do not typically increase digesta viscosity to the same extent as cereal grains [26]. In animal nutrition, NSPs are classified as polysaccharides that cannot be degraded by endogenous enzymes, thus allowing passage to the colon almost completely undigested in monogastrics [27]. These NSPs interfere with nutrient digestibility, decrease phytate dephosphorylation, absorb water, and shorten digesta residence time [28,29]. Not only does the NSP content of plants vary by species or genotype, but also by environmental factors prior to harvest as well as storage conditions following harvest [27]. 

Xylan is a major class of hemicellulose within all cell walls of grasses as well as secondary cell walls of dicots [30]. Xylan is also a major structural element of xylem vessels within stems of plants that aid in the rapid movement of water and also contribute to the thickness of the wall in interfascicular fibers, maintaining mechanical durability [31]. Xylan is a family of structurally diverse NSP that share a β-(1-4)-linked xylopyranose (Xyl*p*) backbone as a common feature [32]. Classifications of xylan are usually based on the degree of substitution and type of side groups attached to the backbone [30,33]. These substitutions are diverse and vary not only phylogenetically but within different tissues and stages of growth within a species [34]. 

Homoxylan is unsubstituted linear polysaccharide that is common in seaweeds of the *Palmariales* and *Nemaliales* order [30]. Glucuronoxylan typically has single 4-O-methyl-α-D-glucopyranosyl uronic acid residues (MeGlcA) attached at position 2 of the main chain of Xyl*p* units [30]. The glucuronic acid side chain, however, may be present in the 4-O-methylated as well as the non-methylated form [35]. In the literature, this configurational type of xylan is generally referred to as 4-O-methyl-D-glucurono-D-xylan (MGX) [30]. Type I primary cell walls of dicots are typically composed of glucuronoxylan with a backbone of β-1, 4 linked xylose substituted with α-1, 2 linked glucuronic acid [36]. These glucuronoxylans bind tightly to glucan chains in cellulose microfibrils and connect to other cellulose microfibrils or other glucuronxylans to help secure cellulose microfibrils into place [37,38]. Both arabinoglucuronoxylan (AGX) and glucuronoarabinoxylan (GAX) are characterized by single MeGlcA and α-L-arabinofuranosyl (α-L-Ara*f*) residue attached at position 2 and 3 of the xylopyranose backbone, also with the possibility of being slightly acetylated [30]. In general, AGX is distinguished by the backbone being substituted to a greater extent by MeGlcA compared to hardwood MGX, with AGX having 5-6 Xyl residues per uronic acid group and MGX averaging around 10 [30]. 

AGX is the major hemicellulose located within cell walls of lignified support tissues of cereals and grasses [39]. GAX is typically found in non-endospermic tissues of cereal grains such as corn, rice bran and wheat and consists of an arabinoxylan backbone characterized by approximately ten times fewer uronic acid side chains than α-L-Ara*f* side chains. The ratio of arabinose to xylose (A:X), content of MeGlcA and the presence of disaccharide chains differ from the source in which they are extracted, and this reflects differences in the degree and pattern of substitution in GAX [39]. Arabinoxylan (AX) is the most common form of xylan found in cereal grains such as wheat, rye, barley, oat, rice, corn, and sorghum [40], and is characterized as having a linear backbone substituted with α-L-Ara*f* residues positioned either on O-2 or O-3 or on both the O-2 and O-3 of the Xyl*p* monomer units [35]. Xyloglucan (XG) is the primary hemicellulose found in dicotelydons, such as soybeans, and is characterized by a glucose back bone with xylose side chains attached to carbon 6 of the glucose residues in the chain [41]. Due to the structure of XG, this polysaccharide possesses a “mucin-like” molecular structure that grants mucoadhesive properties [42]. This allows XG to act as a physical barrier that can protect the integrity of mucosal cells against potentially pathogenic microorganisms, pro-inflammatory compounds and allergens [42]. Heteroxylan is structurally more complex than other xylans and is characterized by being heavily substituted with various mono or oligosaccharides and tends to be present in cereal bran, seed, and gum exudates [43]. With regard to the feeding of livestock, AX and XG are of main interest as these polymers are the most prevalent in common monogastric feeds that utilize a corn/soybean base. 

The cell wall of most cereal grains is composed of 60–70% AX located in the aleurone and endosperm [44]. Within the endosperm, however, the degree and pattern of arabinose substitution and molecular mass differ between wheat [45], barley [46], rye [47], and oat [48]. Jaworski et al. [49] investigated the carbohydrate composition of major feedstuffs and found that 48.65% of the total NSP content of corn consists of AX and 48.7% of the total NSP in corn DDGS consisted of AX. Sorghum, sorghum DDGS, wheat and wheat bran had AX levels of 44.3%, 41.2%, 63% and 64.3% of the total NSP composition, respectively. The amount of insoluble AX, as well as the arabinose to xylose (A:X) ratio, determines how complex the AX structure is, the higher the degree of arabinose substitution, the higher the crosslinking between AX and the lower the enzymatic degradation [50,51,52]. It has long been established that energy obtained from the absorption of pentose sugars, such as xylose and arabinose, is lower when compared to hexose sugars, such as galactose, glucose, and mannose, within the small intestine [53]. Arabinoxylan followed by mannan are the two most common pentose sugars present in cereal grains, such as corn, wheat, and barley, and are considered major antinutritional factors in the feeds presented to monogastric livestock species [54,55]. 

Studies performed in broilers found that AX is not digested in the small intestine and produces a viscous chime in the intestine, leading to proliferation of pathogenic bacteria, intestinal inflammation, and impairment of barrier function in the intestine and severe intestinal lesions [56,57]. Supplementation of enzymes within feeds fed to chickens has demonstrated improved growth performance and nutrient utilization by lowering the gastrointestinal viscosity related to AX and β-glucan ingestion [58,59]. Enzyme supplementation, such as xylanase in feeds fed to swine, has also reported improvement in nutrient digestibility [60], as well as a reduction in digesta viscosity [61]. The use of exogenous enzymes within formulated feeds fed to both swine and poultry is a common strategy to help alleviate some of the antinutritional properties of NSP present in common feedstuffs [60,62,63]. Due to high levels of phytate being generally concentrated in the fibrous cell walls of feedstuffs, mixtures of NSP-hydrolyzing enzymes and phytase have been proposed to further improve nutrient digestibility in swine [61]. For instance, in an in vitro study simulating the monogastric digestive tract, the addition of xylanase increased the availability of soluble minerals in corn (copper, zinc, and manganese), wheat (copper, zinc, iron, and manganese), wheat middlings (zinc), barley (copper and manganese), wheat bran (manganese), and soybean meal (copper) [64]. According to the same authors, such an increase in mineral availability could be obtained by a reduction in the antinutritional factor of xylan by half (on average 55.3%) in feedstuffs after inclusion of xylanase. Polysaccharides such as mannan and AX are considered anti-nutritional compounds due to their detrimental impact on nutrient utilization [65,66], conversely, oligomers produced from AX and other xylan exert a more functional purpose in enhancing monogastric intestinal health.

Although AX is typically viewed as an antinutritional factor present in common cereal grains that must be overcome, the effects of all polymers within the xylan family are not as defined or understood. The mucoadhesive properties of XG allow the compound to act as an additional hurdle for pathogen invasion through the reduction in bacterial adherence as well as having a role in the preservation of tight junctions and paracellular flux in vitro [67,68,69]. XG is a water-soluble polymer that can form a neutral and viscous aqueous solution [70]. Though not much focus has been placed on XG in the feeding of livestock, studies have been conducted on the possible beneficial effects of XG on intestinalmicrobiota. In vivo, it was found that Firmucutes and Bacteroidetes are the two dominant phyla that utilize XG [71]. More specifically, two strains of bacteria, *Clostridia* spp. and *Bacteroides* spp., have been identified in the rumen as being able to utilize XG [72]. A study conducted by Moro Cantu-Jungles [73] demonstrated that the utilization of tucuma pulp XG by human intestinal microbiota altered the composition and abundance of the microbiota, highlighted with an increase in Bacteroidetes and reduction in Firmicutes and Actinobacteria. More specifically, *B. uniformis* was considerably enhanced and *Bifidobacterium* sp. was notably decreased. The majority of studies involved with XG are performed in single animal models with rats serving as the primary model; no further research exploring the mechanism by which XG forms a protective layer in the intestinal tract or the physiological effect of that protective layer is available at this time. More research in this area is needed to solidify the effects that XG has on intestinal health and, consequently, growth performance. The antinutritional effects of xylan on swine and poultry performance can be seen in Table 2. 

## 3. Production of Xylooligosaccharides

Oligosaccharides are short-chain polymers of monosaccharide units connected by α or β glycosidic bonds. Xylooligosaccharides (XOS) are an emerging prebiotic produced through chemical and/or enzymatic processing of lignocellulosic materials (LCMs). During the pretreatment phase of LCMs for production of oligosaccharides, the large majority of insoluble hemicelluloses are separated from the cellulose microfibrils surface and degraded to numerous soluble oligosaccharides. The amount, as well as the structure of the oligosaccharide, is dependent on the method of pretreatment as well as the severity of the extraction process. Different methods of production of XOS include autohydrolysis through heating LCMs in water or steam, chemical treatments in dilute solutions of mineral acids [86], direct enzymatic hydrolysis [87,88], or chemical fractionation to isolate xylan in the LCM, which will later be converted to XOS through enzymatic hydrolysis [89]. 

Xylan extraction accomplished through steam and acid treatments has been shown to produce large amounts of monosaccharides along with their dehydration products [90,91]. Autohydrolysis or the degradation of xylan through the use of steam results in increased production of acetic acid through the deacetylation of xylan, which in turn hydrolyzes hemicellulose present in LCMs [92,93]. Although the autohydrolysis method of extraction avoids the use of corrosive chemicals, which could be perceived as advantageous to consumers, the process requires special equipment that has the ability to operate at high temperatures and can produce large amounts of undesirable by-products. The variety of compounds formed by water or steam treatments includes monosaccharides, acetic acid, lignin fractions of the parent stock, furfural, inorganic components, and protein-derived products [94]. Refinement of the autohydrolysis liquors must be accomplished through the removal of monosaccharides as well as non-saccharide components to obtain a product with the highest XOS concentration as possible [95]. Ethanol, 2-propanol, and acetone have been used in solvent extraction and precipitation for refinement of XOS; these compounds aid in the removal of non-saccharide components within autohydrolysis liquors and result in a fraction that is solvent-soluble, consisting of phenols and extractive-derived compounds as well as the refined aqueous fraction [96]. The degree of purity and recovery yields is not only dependent on the type of solvent used, but also the type of LCMs used, as this determines the XOS substitution pattern and the presence of non-saccharide components [97]. Ethanol has achieved the best purification results but exhibited limited recovery yields [97,98,99]. The production of XOS manufactured by autohydrolysis has been accomplished in a myriad of feedstuffs and biomaterials such as corncobs [100], barley hulls [92], brewery spent grains [101,102], rice hulls [98], corn fiber [103], hardwoods [92], and softwoods [104].

Basic or dilute acidic media can also be used to produce XOS through hydrolytic processes. The most commonly used acid for XOS production is dilute sulfuric acid (0.1–0.5 M) [105]. The concentration of the acid, temperature, and reaction time determines the degree of polymerization (DP) distribution of the XOS formed, the amount of monosaccharides is determined by the structure and composition of xylan in the LCM [86]. The advantage of using acid hydrolysis for the production of XOS is the simplicity as well as the rapidity of the process. Akipinar et al. [86] reported the reaction time of dilute acid hydrolysis took a few minutes in contrast to several hours using enzymatic hydrolysis to produce the same XOS conversion rate. The disadvantage of acid hydrolysis, however, is the low yield of oligomers compared to monomers as well as the production of undesirable byproducts such as furfural [105]. Methods for the removal of these byproducts include membrane separation and adsorption chromatography.

Alkali compounds such as NaOH, KOH, and Ca (OH)₂ or a mixture of these compounds can be used to extract xylan from LCMs, which can then be converted to XOS via xylanase enzymes with low exo-xylanase and/or β-xylosidase activity [88]. Production of XOS through enzymatic hydrolysis has been accomplished using cotton stalks [88], corn residues [106,107], wheat straw [108], oat spelt [109], beech wood [110], and hardwood [100,111]. The benefit of enzymatic hydrolysis is the lower production of undesirable monosaccharides and other by-products compared to autohydrolysis extraction as well lack of special equipment needed for extraction. Enzymatic hydrolysis, however, results in much longer reaction times in comparison to autohydrolysis or acid hydrolysis techniques [105].

Due to the relatively recent interest in the prebiotic industry for scientific research and food applications, the classification of what constitutes a prebiotic is evolving. Gibson et al. [11] suggested that a prebiotic is a selectively fermented ingredient that allows specific changes—both in the composition and/or activity of the gastrointestinal microbiota that confers benefits. Xylobiose is characterized by having two xylose residues in its structure; xylotriose contains three xylose residues, and so on. The amount of xylose residues varies from 2 to 10 in XOS production usually, with the xylan source as well as the method of extraction impacting DP, monomeric units, and types of linkages [95]. These variations can have a large impact on the prebiotic activity of XOS as the DP has been shown to have a significant effect on the bifidogenic ability in the intestine [112]. Xylobiose and xylotriose have been shown to be significantly more effective for the growth of bifidobacteria when compared to longer chains of XOS [112]. This is important as XOS mixtures that differ widely in composition can have very different dosage requirements and selectivity in terms of fermentation. 

## 4. Xylooligosaccharides on Intestinal Health and Performance

The gastrointestinal (GI) tract of pigs and poultry contains the voluminous portion of immune tissue located in the body, encompassing over 70% of the total immune cells in the organism [113]. Such massive presence of immune cells is necessary to provide selective absorption of nutrients in addition to preventing harmful effects of toxins and potential pathogens from the intestinal lumen. The commonly used term “intestinal health” consists of five distinct domains that provide a complex and thorough definition: (1) a balanced feed that provides all required nutrients and energy to the animal; (2) a robust mucosal layer that preserves intestinal integrity; (3) adequate immune responses; (4) motor and neuroendocrine functions of the intestine; (5) a balanced intestinal microbiome that sustains a stable and healthy intestinal environment [113,114,115]. The physical barrier of the GI tract includes epithelial cells, as well as intercellular tight junctions and mucus; the chemical and immunological barrier consists of secretions, such as lysosomes and proteolytic enzymes, as well as gastric acid. Tight-junction proteins within the structural barrier create a seal between two enterocytes and help to regulate intestinal permeability. Mucosal immunoregulation in the intestine is generally controlled by a single layer of epithelial cells that act as a barrier between the contents of the lumen and the lymphocyte-rich lamina propria [116]. These epithelial cells can recognize antigens and bacterial lipopolysaccharides through pattern recognition receptors and respond with various chemokine and cytokine secretions from local intestinal immune cells [117]. Disruption of this structural barrier is known as hyperpermeability or leaky gut syndrome [117]. 

When intestinal health is threatened by immunostimulatory components and/or exogenous polysaccharides residing within the lumen, feed efficiency and growth performance suffer, ultimately causing depressed economic returns [115]. Supplementation of oligosaccharides is one strategy that can aid in the reduction in immune response stimulation and help mitigate the adverse effects on intestinal health caused by polymeric forms within the dietary fiber source [53]. XOS resists salivary hydrolysis as well as hydrolysis catalyzed by gastric and pancreatic secretions, allowing passage through the small intestine to the colon of pigs or ceca of poultry, providing a substrate for microbial fermentation [118]. The functional difference between the oligosaccharide and polysaccharide form of xylan is the inability of XOS to form cross-linkages and stimulate immune receptors [115]. Therefore, it is speculated that XOS may help to reduce stimulation of the immune response in monogastric animals, thus limiting the metabolic cost associated with maintaining that immune response. Recent studies investigating the impact of XOS supplementation on swine and poultry growth and health can be found in Table 3.

Host and intestinal microbes constantly interact with one another resulting in modulation of various physiological responses and functions of the host. Functional oligosaccharides derived from xylan have been demonstrated in several studies to alter the microbial ecology in the intestines of monogastric animals. In a study conducted by Okazaki et al. [112], the authors utilized a mixture of XOS with varying DP: xylose (DP:1), xylobiose (DP:2), xylotriose (DP:3), as well as other saccharides for in vitro fermentations with *Bifidobacterium adolescentis*, *Bifidobacterium infantis*, and *Bifidobacterium longum* along with other microorganisms. The authors reported that *B. adolescentis* showed exceptional ability to utilize both xylobiose and xylotriose. The XOS mixture composed of mainly xylobiose was greatly utilized by *B. adolescentis,* as well as *B. infantis* and *B. longum*. Lactobacillus species were found to only slightly utilize XOS, except for *L. fermentum*. The authors also concluded that *Staphylococcus* and *E. coli* did not utilize XOS as an energy source but utilized glucose well, whereas most clostridium species did not utilize XOS. The authors then conducted an in vivo experiment with XOS in humans and found that Bifidobacteria counts increased from 10% (pre-administration of XOS) to 32% by the 2nd week following the onset of XOS supplementation. The XOS-supplemented group also experienced decreased pH of the feces and maintained the water content of the feces within a normal range, both contributing to the establishment of a suitable condition for the intestine. These results were obtained by providing daily supplementation of just 2 g of XOS. 

Furthermore, a study performed by Smiricky-Tjardes et al. [119] investigated fermentation characteristics of different oligosaccharides by swine fecal microbiota. The oligosaccharides that were evaluated included short, medium, and long-chain fructooligosaccharides (FOS), stachyose, raffinose, transgalactooligosaccharides, glucooligosaccharides (GOS), mannanoligosaccharides (MOS), and xylooligosaccharides. The authors reported that fermentations of XOS resulted in the lowest pH at 8 and 12 h followed by GOS. These data indicate that bacteria present in the large intestine may be able to ferment the XOS substrate more extensively compared to the other oligosaccharides tested. In addition, XOS fermentation produced the highest total short chain fatty acid (SCFA) concentration among all oligosaccharides tested. The importance of SCFA production should never be underestimated as SCFA has been found to contribute up to 28% of the total maintenance energy of pigs [120]. In a study conducted by Finegold and others [121], bifidobacteria counts in the human intestine increased 21% at 4 weeks from baseline and 17% at 8 weeks from baseline in the high dose XOS group (2.8 g/d). The study also reported increased numbers of *B. fragilis* in the high dose group; however, no increase in lactobacilli was observed. At the genus level, *Faecalibacterium* spp. and *Akkermansia* spp. were significantly increased in subjects consuming the high dose of XOS. *Faecalibacterium* spp. is a butyrate-producer and has known anti-inflammatory properties within the intestine [122]. *Akkermansia* is a mucin-degrading bacterium that has been associated with improved intestinal health [123]. Jaskari et al. [124] and Van Laere et al. [125] both found that XOS sourced from oats was not exclusively fermented by bifidobacteria but also, *Bacteroides* spp., *Lactobacillus acidophilus*, *Klebsiella pneumoniae* and *Clostridium* spp., all reporting moderate growth using this substrate.

In a study conducted by Pan et al. [126], the authors evaluated XOS supplementation on the microbial activity and concentration in grow-finish phase pigs. The authors found that supplementing 100 g/ton XOS during the grow-finish phase of production led to an increase in the relative abundance of lactobacilli, as well as an increase in SCFAs and bioamines. The levels of acetic acid, propionic acid, and SCFA were significantly higher in the 100 g/ton XOS group than the non-supplemented group; the author suggests this points to certain bacteria, such as lactobacilli, having a key role in SCFA production. Compared to the antibiotic-fed group (0.04 kg/ton virginiamycin and 0.2 kg/ton colistin), the XOS supplemented group had significantly improved the relative abundances of bacteria often considered beneficial such as Coprococcus, Lactobacillus, Roseburia, and Ruminococcus in addition to increased luminal concentrations of SCFAs, which are considered advantageous for intestinal health [127]. In a similar study, Liu et al. [128] investigated the effects of probiotic or xylooligosaccharide supplementation on nutrient digestibility, noxious gas emission, and intestinal health in weanling pigs. The authors reported that XOS supplementation of 200 mg/kg for 28 days improved both ADG (17%) and G:F (14%) in weanling pigs also in addition to lowering the fecal NH₃ concentration. Conversely, Yin et al. [129] reported that XOS supplementation had no effect on growth performance or biochemical parameters in weaned pigs but did markedly enhance the α-diversity of the intestinal microbiota. The author partially attributes the discrepancy of growth performance on the low dosage (0.01% XOS) used in the study and called on future studies to confirm the optimal dose of XOS for weaned pigs.

Madhukumar and Muralikrishna [130] evaluated the ability of different strains of *Bifidobacteria*, *Lactbacilli*, and *Pediococci* spp. to utilize XOS from Bengal gram husk (BGO) and wheat bran (WBO). All bacterial strains tested were found to readily use XOS; this was concluded due to the increase in turbidity of the culture broth, xylanase, xylosidase, and arabinosidase activity, dry cell mass and the increased production of short chain fatty acids. Out of all the microbes tested, *Lactobacillus brevis* NDRI strain RTS and *Pediococcus pentosaceus* NCDO 813 more effectively utilized XOS from both WBO and BGO. These two microorganisms were followed by *Bifidobacterium adolescentis* NDRI 236, *Bifidobacterium bifidum* ATCC 29521, *Bifidobacterium bifidum* NCDO 2715, *Pediococcus pentsaceus* ATCC 8081 and *Lactobacillus plantarum* NDRI strain 184. These findings are in line with previous studies confirming that Bifidobacterium strains are able to efficiently ferment xylose-based oligosaccharides [118,131]. Beneficial bacteria such as Bifidobacteria and lactic acid bacteria produce enzymes that degrade carbohydrates and ferment non-digestible oligosaccharides; this leads to the production of SCFAs that provide energy to the host as well as aids in the acidification of the bowel [132]. The increase in production of SCFAs as a result of fermentation also decreases intestinal pH, which correlates with increased population growth of beneficial microbes thus inhibiting the growth of potentially pathogenic bacteria [133].

Lin and others [134] conducted a randomized controlled study evaluating the prebiotic effects of XOS on fecal microbiota in healthy human volunteers after a period of 6 weeks (1.2 g XOS/d). The results showed that XOS supplementation significantly increased fecal bacteria counts of *Bifidobacterium* spp., *Lactobacillus* spp., as well as decreased counts of *Clostridium perfringens* without altering the total anaerobic bacterial counts. *Clostridium perfringens* is considered to be detrimental as it is an opportunistic pathogen with the ability to cause food poisoning and necrotic enteritis [135]. The authors attribute the decreased counts of *Clostridium perfringens* to the increased production of SCFA and consequently the decrease in intestinal pH. Hsu and others [136] evaluated XOS, as well as FOS supplementation on cecal microbiota, cecal pH, cecal weight, serum lipid levels, and their inhibitory effect on precancerous colon lesions in male rats. The researchers found that supplementation of 60 g/kg of XOS or FOS for 35 days significantly increased the intestinal bifidobacteria populations as well as decreased cecal pH levels when compared to the control. The XOS supplemented group had greater colonic wall and cecal wall relative weights as well as a greater bifidobacteria population than the FOS supplemented group. The authors attribute the increased cecal total and wall weights to a normalization of epithelial cell proliferation due to the increased production of SCFA. Howard et al.’s [137] findings were in accordance with the previously mentioned study and reported that XOS supplementation increased cecal wall density due to an enhancement in epithelial cell proliferation. A previous study conducted by Tomomatsu [138] reported the effective daily dose of FOS in humans is 3.0 g/d, yet only 0.7 g/d for XOS, suggesting XOS may be more effective than FOS. Additional effects of XOS supplementation include improvement of bowel function, calcium absorption, and lipid metabolism along with lowering of cardiovascular disease and colon cancer [139]. XOS has also been shown to be thermostable during pasteurization and can be autoclaved at a lower pH than FOS, which is found to be more susceptible to degradation at higher temperatures and lower pH [140]. This is significant as XOS should theoretically be more resistant than FOS to common feed milling processes such as forms of extrusion and pelleting in addition to possessing easier storage options.

## 5. Xylanase 

In addition to chemical and enzymatic treatments of LCM for the production of XOS to be used in animal feeding, supplementation of xylanase in feeds offered to monogastric animals is also of great interest. As previously stated, common cereal grains and related coproducts contain fluctuating amounts of NSP which can increase the viscosity of the digesta in the small intestine resulting in the reduction in digestibility and absorption of nutrients. Furthermore, the increase in digesta viscosity can lead to morphological changes on the mucosal surface [4,24], increased pathogenic load in the small intestine [148] and increased digesta transit time [149].

Xylanases are classified as glycosidases that catalyze the hydrolysis of 1,4-β-D-xylosidic linkages in the polymer xylan [1]. Endo-xylanases are characterized by hydrolyzing bonds in the interior of xylan polymers or exo-xylanases that act by hydrolyzing xylan from either the reducing or non-reducing end [150]. Originally, Wong et al. [150] attempted to classify xylanases based on their physicochemical properties and suggested two classification, xylanases with low molecular weight (<30 kDa) and basic p*I*, and xylanases with high molecular weight (>30 kDa) and acidic p*I*. This was soon replaced by a more complete system [151] as approximately 30% of identified xylanases, specifically fungal xylanases, could not be classified under this system [1]. This novel classification system not only facilitated the classification of xylanases but glycosidases in general (EC 3.2.1.x) and is still the standard means of classification to this day. This system classifies enzymes based upon the primary structure of the catalytic domain only and then categorizes enzymes of related sequences into families [152,153]. Initially, xylanases and cellulases were grouped into six families; today there are 168 glycoside hydrolase (GH) families recognized on the CAZy database (http://www.cazy.org/, accessed on 22 Decmber 2020). Due to divergent evolution, some families have related three-dimensional structures and thus are grouped into higher hierarchical levels known as clans [154]. To date, 18 different clans have been proposed (GH-A to GH-R) with the majority of clans containing two to three families aside from GH-A which contains 24 families. Xylanases are typically reserved to families 10 and 11 [155,156,157,158]. 

Up to now, GH5, GH8, GH10, GH11, GH30, GH43, GH51, and GH98 are the eight glycoside hydrolase families that EC 3.2.1.8 is classified under [149]. The classification of different endo-xylanases is reflective of their use in various applications with GH10 and GH11 being the most commonly used and researched. Both fungal and bacterial from GH10 and GH11 constitute the predominate enzymes used in trials for the production of different types of XOS [149]. The genes obtained from bacterial endo- β-xylanases are mainly from Gram-positive bacteria under the phyla Firmicutes and Actinobacteria with the classes Bacilli and Clostridia being the most commonly derived from Firmicutes [149]. The genus, Streptomyces, constitutes the majority of candidates from Actinobacteria derived endo- β-xylanases [159,160,161]. The pattern of XOS obtained has been found to be at least, to some extent, GH-family dependent. This is highlighted by a study performed on *Trichoderma* sp. xylanases, where GH10 xylanases were shown to have higher activity on smaller substrate molecules resulting in the preferential production of XOS with lower DP (xylobiose to xylopentaose). Conversely, GH11 xylanases were more active on larger substrates and resulted in XOS with higher DP (xylobiose to xylohexose) [161]. In addition, Abou-Hachem [161] found that two xylanases from *Psedozyma hubeiensis*, representing both GH10 and GH11, produced no xylose or xylobiose but only produced xylobiose to xyloheptose. This finding indicates that the pattern in which XOS is produced is also enzyme dependent and not solely family dependent. GH10 xylanases have been shown to hydrolyze β-1,4-xylosidic linkages in heteroxylans but can also handle glucose to some extent when in the active site [149]. GH10 xylanases have been reported to have low ability to act on insoluble forms of xylan [144] but can act on substitutions to the xylose backbone. Interestingly, GH10 xylanases have shown high affinity for short XOS indicating a small substrate binding site is present [149].

Enzymes in GH11 show high activity for heteroxylans with a backbone of xylose units and absence of glucose. Interestingly, GH11 enzymes prefer longer substrates with the affinity for XOS increasing from DP 3 (xylotriose) up to DP 5 (xylopentose) and no activity on XOS with a DP of less than 3 [162]. The restrictions of substitutions for GH11 enzymes typically result in total degradation of substituted xylan being lower compared to GH10 enzymes, which can handle a higher degree of substitutions at the active site [163]. Due to the smaller size of GH11 xylanases, however, the enzyme is shown to be more efficient in degrading insoluble xylan compared to GH10 xylanases [164]. 

Enzymes previously assigned to GH5 have now been reclassified to GH30 as phylogenetic analysis showed these enzymes were more similar to GH30 [165]. GH30 enzymes are grouped into eight subfamilies, predominantly based on the arrangement of secondary structures that form the side β-structure [149]. The enzymes with the majority of the xylanase activity reside in subfamily 8 with the only exception being two characterized xylanases in subfamily 7 [166,167]. As of now there are nine characterized GH30 xylanases that are structurally determined with four having been extensively researched, BSXynC from *Bacillus subtilis* [167,168], EcXynA from *Erwinia chrysanthemi* [169,170], Xyn30D from *Paenibacillus barcinonensis* [171], and CpXyn30A from *Clostridium papyrosolvens* [172]. Both BsXynC and EcXynA were the first xylanases classified as GH30 and characterize the common features seen with GH30_8 xylanases [149]. Both of these xylanases differ from those found in GH10 and GH11 due to high affinity for glucuronoxylan and xylooligosaccharides substituted with methylglucuronic acid or glucuronic acid and very low affinity for unsubstituted xylan, XOS and arabinoxylan [149]. Xyn30D from *Paenibacillus barcinonensis* is solely assigned to EC 3.2.1.136 and is exclusively active on glucuronoxylan [171] and CpXyn30A from *Clostridium papyrosolvens* is solely designated to EC 3.2.1.8 and shows moderate affinity for glucuronoxylan yet high affinity for arabinoxylan and XOS [172].

β-d-xylosidases (EC 3.2.1.37.) cleave monomers from the non-reducing end of xylooligosaccharides; however, acetylxylan esterases (EC 3.1.1.72), ferulic acid esterases (EC 3.1.1.73), α-l-arabinofuranosidases (EC 3.2.1.55.), p-coumaric acid esterases (EC 3.1.1.B10), and α-d-glucuronidases (EC 3.2.1.139) catalyze the removal of side groups [1]. Thus, in theory, supplementing xylanase in corn-based feeds, an ingredient rich in fiber, should enhance the energetic contribution of said fiber resulting in improved growth performance. However, very few studies report an improvement in both digestibility and performance [173]. A hypothesis by Bedford [174] proposed that rather than non-starch polysaccharide enzymes (NSPases) producing more fermentable sugars, these NSPases may produce oligosaccharides that then signal to the microbiome and increase the capacity of the entire system to degrade fiber. According to the author, this is evidenced by a recent study performed in chickens that revealed 35 d of xylanase supplementation resulted in greater fermentation of pentoses and AXOS in the cecum of supplemented birds compared to the control. In a study conducted by Duarte et al. [5], newly weaned pigs were supplemented with a xylanase or a protease or a combination of both throughout two feeding phases for a total of 24 days. It was found that the BW of pigs increased when both enzymes were used in conjunction but not when individually supplemented. At the culmination of the trial, it was concluded that xylanase supplementation (45,000 XU/kg) improved the growth performance and intestinal morphology, reduced digesta viscosity and reduced intestinal oxidative stress in newly weaned pigs; however, the protease was shown to be more effective in the maintenance of intestinal morphology due to increases in villi length and reductions in crypt depth and cell proliferation. These results correlate nicely with the findings of Passos et al. [4], who observed that xylanase supplementation reduced jejunal digesta viscosity in nursery pigs when fed feeds consisting of 30% DDGS, thus enhancing nutrient utilization. Data obtained in the study also indicated that as the dietary level of xylanase increased, digestibility of DM OM, energy, NDF and crude ash increased by 9.2, 8.5, 9.3, 12.4, and 10.7%, respectively. These results are in accordance with an earlier study conducted by Diebold et al. [175] as increases in ileal digestibility of OM, CP, CF, NDF, and energy resulted from xylanase supplementation in nursery pigs, however, to a lesser extent. Interestingly, a study focused on the supplementation of xylanase in the feeds offered to growing pigs (26 kg) found that improvements in AID of DM, CP, and energy were small (<2%) but significant [176]. Naturally, mature pigs have a larger and more developed GIT with higher cellulolytic activity as well as lower feed intake per kg of body weight and slower digesta transit time [177]. This may be attributed to the ability of older and more mature pigs, such as sows, to digest a greater amount of fibrous components in comparison to young pigs, such as nursery or early growers, as fiber digestibility and the capacity for fiber degradation increase with body weight [178]. Indeed, Jørgensen et al. [177] reported that sows digest a larger portion of NSP in the small intestine compared to growing pigs. The authors also found that sows had a higher capacity to digest insoluble NSP; however, no such difference in digestibility of soluble NSP between sows and growing pigs was observed. This enhancement in fiber degradation and capacity in more mature and heavier weight pigs may limit the effectiveness of xylanase supplementation when added to feeds. A trial conducted by Kerkaert et al. [179] investigated the effects of xylanase on growth performance as well as carcass characteristics in 1944 mixed sex growing-finishing pigs (initial BW: 22.63 kg, final BW: 133.64 kg) and found that, overall, there was no difference in ADG, ADFI, or FCR between the supplemented pigs and control. It is interesting to note that the authors used increasing levels of xylanase in six nutritionally adequate corn-soy based feeds at levels of 0, 2.3, 4.5, 9.1, 18.1 and 34.0 SXU/lb of enzymatic activity for xylanase (IU)/lb (0, 5, 10, 20, 40, 75 IU/kg) of added xylanase. Although no difference was found in growth performance or percentage of pigs receiving injectable treatments and overall mortality, carcass yields did increase when intermediate levels of xylanase were fed. 

Xylanase inhibitors in common feedstuffs have proven to be yet another obstacle to overcome. The presence of these inhibitors was first discovered in the late 1990s within wheat [180,181]. Following the initial discovery, these proteinaceous xylanase inhibitors were found in barley [182], rye [183], and maize [184]. Wheat (*Triticum aestivum*) was found to contain three structurally different inhibitors, *T. aestivum* xylanase inhibitor (TAXI) [180], thaumatin-like xylanase inhibitor (TLXI) [185] and xylanase-inhibiting protein (XIP) [186]. Other cereals with proteinaceous inhibitors of xylanase are similar in structure to both XIP and TAXI found in wheat thus are often called XIP-like and TAXI-like proteins [183]. Studies have shown that these inhibitors can specifically affect microbial xylanases belonging to GH families 10 and 11 but, interestingly, have no effect on endogenous enzymes synthesized by plants and xylanases outside of the previously mentioned families [183,184,185,186,187]. There have been specific microbial xylanases from GH families 10 and 11 that are insensitive to proteinaceous inhibitors, but these are thought to be the exception and not the rule. 

XIP-I has been found to competitively inhibit fungal xylanases in GH families 10 and 11 but not to the same extent in bacterial xylanases found in these families [183,186]. XIP-I has also been found to inhibit α-amylases in GH family 13 [188,189] and barley α-amylase/subtilisin inhibitor (BASI) has been shown to partially inhibit xylanase in GH family 11 [189]. This is noteworthy as some authors suggest this cross inhibition of xylanases and amylases by proteins from cereals may have developed throughout the plants’ evolution as a protective function against external attack of phytopathogens [182]. XIP-like inhibitors have been found in barley [190,191], maize [191], rice [192,193], oat [194], and Algerian pearl millet [195]. TAXI-like proteins typically inhibit fungal and bacterial xylanases from GH family 11 but not GH family 10, with TAXI-I-like proteins generally inhibiting xylanases with low and high p*I* values and TAXI-II-like proteins solely inhibiting xylanases with high p*I* [188,196]. TLXI is the newest classification of proteinaceous xylanase inhibitor, being discovered in 2006 [194]. Similarly, TLXI proteins tend to inhibit xylanases from GH family 11 but have no such effect on GH family 10 [184]. Data are limited regarding the presence of TLXI-like inhibitors in other cereals besides durum wheat [194]. The TAXI, XIP, and TLXI content in cereal grains is highly variable and dependent on the breed, cultivar, time of harvest as well as the specific fraction of the grain [195,196,197]. For example, the XIP-I content of wheat belonging to the same cultivar, ranged from 0.12 to 0.6 mg/g in the flour and 0.21–0.56 mg/g in the grain [197]. TAXI content of 20 different cultivars of wheat grain grown in France saw variation from 0.05 to 0.19 mg/g [197]. The response to xylanase supplementation in swine and poultry feeds can be seen in Table 4. 

## 6. Conclusions

In conclusion, understanding the structural difference, type, and concentration of xylan within the plant material, as well the various xylanases available for use, is paramount for the future of feeding animals and understanding the role of fiber within feeds. Characteristic differences in xylan substrates have a direct impact on both endogenous and exogenous cleavage of the polymer to more functional oligosaccharides and thus are central in giving rise to the desired prebiotic effects. Supplementation of XOS seems to confer physiological benefits in various animal species and typically exerts these benefits at a lower dose than other prebiotics. Available experimental evidence supports the claim that XOS can help prevent or mitigate gastrointestinal disorders as well as increase the numbers of beneficial bacterial populations. These results are encouraging; however, data on XOS are still limited in comparison to other prebiotics, such as FOS, MOS, and GOS, so additional studies are needed to solidify the promising initial results. The supplementation of xylanase within feeds fed to poultry and swine arose to combat the antinutritional effects of NSP in common feedstuffs and provides an opportunity to improve the energetic contribution of fiber and produce XOS for fermentation in the distal intestine. However, the response to xylanase supplementation is highly variable due to a number of factors such as age of animal, feed composition, duration of supplementation and presence of xylanase inhibitors. Additionally, even when benefits are reported with xylanase supplementation, whether those benefits are a result of nutrient release from NSP, reduction in digesta viscosity or production of short chain xylooligosaccharides is still in question. As with many other feed additives, the effects, as well as the mode of action, are likely multifactorial. Issues related to digesta viscosity are mainly associated with the feeding of poultry; however, the poultry industry is much more consolidated in terms of genetic variability and diet composition. As the swine industry progresses in becoming more sustainable to feed the growing global population, more coproducts from various industries may be implemented into swine feeds, thus necessitating the for understanding the role that digesta viscosity plays in the feeding of these animals. Future studies focusing on the in-vivo mode of action of xylanase in corn-based feeds fed to poultry and swine could greatly improve the consistency of feeding outcomes as well as our understanding of carbohydrases in general. Whether providing xylanase to the feeds to hydrolyze xylan within the intestine of the animal or supplementing XOS prepared in-vitro to the feeds of animals for improving growth rate and intestinal health is still up for debate and future studies should focus on this gap in scientific knowledge. Comparisons among and within species on the extent of physiological effects that XOS can confer is clouded not only by differences in purity of the XOS product administered, but also by the degree of polymerization and type of substitution present in the product. 

## Figures and Tables

**Table 1:** Relative non-starch polysaccharides (NSPs) in feedstuff ^1^.

Feedstuff	Arabinose, %	Xylose, %	AX, %	β-Glucan, %	Cellulose, %	Total NSP, %	Reference
Corn grain	1.2 (0.5)	1.7 (0.4)	ND	ND	1.7	8.1 (2.5)	[49]
	ND	ND	5.1 (0.1)	ND	2.0	8.0 (0.1)	[28]
	ND	1.7	4.3	0.3	2.0	8.3	[74]
	2.0 (0.3)	2.7 (0.2)	4.7 (0.5)	0.1	2.0	9.0 (1.1)	[75]
Soybean meal	1.7 (0.9)	1.7 (0.2)	ND	ND	6.2	21.7 (6.3)	[76]
	ND	ND	0.4	0.7	5.9	25.7	[74]
	2.6 (0.9)	1.7 (0.2)	ND	ND	5.9	21.0 (5.8)	[75]
	3.1 (0.53)	1.7 (0.11)	ND	ND	4.4	19.2 (2.7)	[77]
Wheat grain	ND	ND	7.1	0.6	1.8	10.0	[74]
	1.7 (0.6)	2.9 (0.7)	ND	ND	1.3	9.5 (1.9)	[49]
	ND	ND	6.3 (1.8)	0.4 (0.4)	2.0	9.0 (2.4)	[78]
Sorghum	ND	ND	3.7	0.1	1.1	5.5	[74]
	1.6 (0.1)	1.3	ND	ND	1.5	6.6 (0.4)	[49]
Barley	ND	ND	7.1 (0.8)	0.7 (3.6)	3.9	12.2 (4.5)	[78]
	1.7 (0.3)	2.4 (0.4)	ND	4.2	1.0	12.4 (5)	[76]
Corn DDGS	ND	ND	11.7	ND	10.7	25.3	[74]
	4.3 (0.5)	6.2 (0.9)	ND	ND	5.8	25.0 (3.4)	[49]
	7.9 (2.4)	10.2 (2.6)	ND	ND	6.0	31.8 (8.3)	[24]
Wheat DDGS	ND	ND	12.2	0.3	3.7	17.0	[74]
Rolled Oats	0.6 (0.4)	1.1 (0.2)	ND	4.2	0.6	9.1 (4.8)	[76]

^1^ Values reported on a % dry matter basis. DDGS, dried distiller grains with solubles. AX, arabinoxylan. ND, no data. Values inside parentheses indicated soluble portion of NSP. Values outside parentheses denote insoluble portion of NSP.

**Table 2:** Effects of dietary arabinoxylan content on monogastric animal performance.

Animal	BW	Age, d	Duration, d	Xylan Content	Effect	Reference
Broiler	ND	1	35	0.8% (additional AX in crumble feed) 0.7% (additional AX in pelleted feed)	Increased ileal viscosity, total AX solubilization (22%) and fermentation.	[79]
Broiler	38 g	1	14	2.1% AX (soluble)	Lowered body weight (24%). Increased bifidobacteria population (64%).	[80]
Broiler	ND	6	19	2.5, 5.0, 7.5, 10.0, 15.0% D-xylose	Linear decrease in BWG (5–22%) and FCR (1–10%).	[81]
Pig	6.9 kg	26	30	2.6% AX	Lowered cecal pH (10%). Reduced intestinal transcellular permeability in SI and midcolon (76%, 77%). Increased total SCFA in cecum and midcolon (32%, 19%). Growth performance unaffected.	[82]
Pig	7.5 kg	30	20	4.6% AX	Lowest SCFA concentration. Highest jejunal viscosity. Lower villus height and proliferation.	[24]
Pig	10.7 kg	42	21	1.9% additional AX in feed ^1^	Increased jejunal viscosity (22%) and TNF-α (12%). Reduced AID of DM (8%) and GE (8%). Reduced villus height/crypt depth ratio in duodenum (12%)	[83]
Pig	45–120 kg	ND	ND	12.3% AX	No effect on growth performance. Decreased AID of CP (16%), starch (5%), OM (35%), and energy (20%).	[84]
Pig	58 kg	ND	11	11.8% AX in high fiber diet	Decreased AID of OM (7%), starch (7%), and CP (3%). Decreased AID of Arg (4%), Asp (19%), Glu (3%), Leu (9%), and Ser (12%).	[85]

ND, no data. AX, arabinoxylan. BWG, body weight gain. FCR, feed conversion ratio. SI, small intestine. SCFA, short-chain fatty acid. TNF-α, tumor necrosis factor-α. AID, apparent ileal digestibility. DM, dry matter. GE, gross energy. CP, crude protein. OM, organic matter. ^1^ Calculated according to corn DDGS AX values given by Tiwari et al. [24] and corn AX values given by Knudsen [75].

**Table 3:** Effects of the addition of xylooligosaccharide (XOS) to swine and poultry feeds.

Animal	BW, kg	Age, Day	Duration, Day	% XOS in Feed, (Purity)	Effect	Reference
Broiler	ND	1	42	0.0025, 0.0050, 0.0075, and 0.0100 (35% XOS)	Linearly improved FCR (4%) (100 mg/kg) and water-holding capability in thigh muscle (37%) (100 mg/kg). Linearly decreased duodenal crypt depth (12%) (100 mg/kg)	[141]
Broiler	ND	1	39	0.20 (d 1 to 13) and 0.50 (d 14 to 39) (35% XOS)	Improved FCR (2%). Increased villus length in ileum (14%). Higher abundance of butyrate producing species and *Lactobacillus crispatus*	[142]
Broiler	ND	1	59	0.005, 0.01, and 0.02 (8.24% XOS)	Improved ADG (9.44%), FCR (4.18%) [10 g/kg].Enhanced endocrine metabolism and improved immune function	[143]
Broiler	ND	1	21	0.50 ^2^	Decreased glucose, VLDL, and Streptococci and *E. coli* populations. Increased Bifidobacteria	[144]
Broiler	ND	1	29	0.025 and 0.100 (35% XOS)	0.100% reduced FI (11.82%). Improved FCR (11.5%)	[145]
Broiler ^1^	ND	21	21	0.025 ^2^	Improved BWG (6.01%), G/F (2.32%). Increased proportion of SCFAs	[146]
Laying hen	ND	196	56	0.01, 0.02, 0.03, 0.04, and 0.05 ^2^	Increase in eggshell thickness, eggshell relative weight and plasma 1,25(OH)_2_ D3. Lowered plasma GPT, cholesterol, HDL, VLDL	[147]
Pig	6.30	21	28	0.02 (50% XOS)	Improved ADG (17%), FCR (14%). Decreased NH_3_ levels	[128]
Pig	7.44	ND	28	0.01 (40% XOS)	Reduced serum IFN-γ. Enhanced α-diversity	[129]
Pig	30.00	70	30 to 100 kg BW	0.01, 0.025, and 0.05 (35% XOS)	Increased abundance of Lactobacilli and other beneficial bacteria. Increased SCFA concentration [100 g/t]	[126]

^1^ Challenged with coccidia. ^2^ XOS purity not reported. ND, no data. FCR, feed conversion ratio. ADG, average daily gain. VLDL, very low-density lipoprotein. FI, feed intake. BWG, body weight gain. SCFA, short-chain fatty acid. GPT, alanine aminotransferase. HDL, high-density lipoprotein. IFN-γ, interferon-γ.

**Table 4:** Effects of xylanase supplementation in swine and poultry feeds.

Animal	BW	Age, Day	Duration, Day	Xylanase Activity	Effect	Reference
Broiler	45 g	1	35	1875, 3750, 5625 XU/kg	Linear increase in BWG (2%), AID of DM, CP and GE, VH:CD, *Lactobacillus* populations in ileum and cecum. Decreased FCR (3%) and cecal *E. coli* populations (15%).	[198]
Broiler	ND	1	42	16,000 BXU/kg	Decreased ileal digesta viscosity (19%) and lactic acid in cecum. Increased soluble arabinose and xylose residues in ileum, increased *Bifidobacterium* spp, acetic and butyric acids in cecum.	[199]
Broiler	ND	1	24	16,000 BXU/kg	Improved BWG (2%) and reduced FCR (3%) and jejunal digesta viscosity (30%).	[200]
Broiler	ND	1	28	16,000 BXU/kg	Increased DM retention, NEp (3%), energy retained as fat (5%) and efficiency of energy retained as fat (6%).	[201]
Broiler	ND	1	40	12,000, 24,000 BXU/kg	No difference in growth performance or total VFA concentration.	[202]
Broiler	ND	1	49	16,000, 32,000 BXU/kg	Improved FCR (6%). Improved AID of energy in wheat-based diet. 32,000 BXU/kg increased isovaleric and caproic acid content.	[203]
Broiler ^1^	ND	1	21	16,000 BXU/kg	Increased FI (8%) and BWG (12%). Improved FCR (2%).	[204]
Broiler ^2^	ND	1	21	5500 XU/kg	Increased VH:CD in jejunum (4%). Decreased plasma endotoxin levels. Increased occludin mRNA expression in ileum (22%).	[205]
Broiler	ND	1	42	16,000 XU/kg	No effect on BWG, FI, FCR or carcass traits. Increased serum insulin. Interaction with dietary ME on serum peptide YY concentration.	[206]
Broiler	ND	1	49	16,000 BXU/kg	Birds fed reduced energy diet supplemented with xylanase had performance equivalent to control group. Improved FCR during grower and finisher phase (7%).	[207]
Pig	7.2 kg	21	24	45,000 XU/kg	Increased ADG (6%). Reduced viscosity of jejunal digesta (13%), mucosal MDA (17%), crypt depth (10%) and crypt cell proliferation (15%).	[5]
Pig	7.2 kg	28	28	4000 XU/kg	Improved ATTD of DM (2%), energy (8%), NDF (23%), ADF (19%), hemicellulose (25%), and phosphorus (29%).	[208]
Pig	7.5 kg	23	20	1500 EPU/kg	Increased concentration of SCFA (20%), villus height in duodenum (8%), proliferation rate in crypt of jejunum. Reduced viscosity of jejunal digesta (26%).	[24]
Pig ^3^	7.9 kg	21	20	10,000 XU/kg	Increased ADG in phase 1 (44%), villus height (13%) and VH:CD ratio (22%). Reduced crypt depth and Ki-67^+^ (7%).	[6]
Pig	10.7 kg	42	21	1500 EPU/kg	Increased ADG (7%), AID of GE (7%) and NDF (14%), crypt depth in duodenum (11%). Reduced viscosity of jejunal digesta (14%) and plasma TNF-α (36%).	[83]
Pig	14.2 kg	49	35	8000, 16,000, 32,000 BXU/kg	No effect on growth performance, nutrient digestibility, VFA concentration or peptide YY concentration.	[209]
Pig	17.6 kg	ND	10	700, 1400 LXU/kg	Enhanced ileal digestibility of NDF, DM (14%), OM (13%), and energy (14%) (1400 LXU/kg). Reduced viscosity of jejunal digesta (14%) (700 LXU/kg).	[4]
Pig	24.3 kg	ND	21	450, 900, 1800 XU/kg	Improved ADG (14–16%), AID of DM (2–4%), histidine, and glutamic acid. Increased fecal and ileal *Lactobacillus* populations. Reduced fecal and ileal *E. coli* counts.	[210]
Pig	25.8 kg	ND	16	24,000 BXU/kg	Increased AID of GE (15%) and NDF (41%) in dry wheat diets. Increased AID of NDF (32%) in liquid DDGS diets. Increased crypt depth (14%) of jejunum in DDGS-fed pigs.	[211]
Pig	34.8 kg	ND	14	16,000 BXU/kg	Improved ATTD of GE (1%), DM (1%) and TDF (10%)	[212]

^1^ 1500 FTU/kg of phytase added, diet deficient in Ca (0.20%), available phosphorus (0.18%), ME by 80 kcal/kg and amino acids (5%). ^2^ Challenged with Clostridium perfringens. ^3^ Challenged with ETEC, supplemented with probiotic *Bacillus* sp. 2 × 108 CFU/kg. XU, the amount of enzyme that releases 1 micromole of reducing moieties from 1.5% arabinoxylan substrate solution per minute at pH 5.0 and 40 °C. BWG, body weight gain. AID, apparent ileal digestibility. DM, dry matter. CP, crude protein. GE, gross energy. VH:CD, villus height to crypt depth ratio. FCR, feed conversion ratio. ND, no data. NEp, net energy for production. VFA, volatile fatty acid. BXU, one BXU is defined as the amount of enzyme that produces 1 nmol reducing sugars from birchwood xylan in 1 s at 50 °C and pH 5.3. FI, feed intake. ME, metabolizable energy. ADG, average daily gain. MDA, malondialdehyde. ATTD, apparent total tract digestibility. NDF, neutral detergent fiber. ADF, acid detergent fiber. EPU, one EPU is the amount of enzyme which releases 0.0083 μmol of reducing sugars (xylose equivalent) per minute from oat spelt xylan at pH 4.5 and 50 °C. SCFA, short-chain fatty acid. TNF-α, tumor necrosis factor-α. LXU, one LXU is defined as the amount of enzyme which releases 1 μmol of reducing sugars equivalents (as xylose or glucose) from birch xylan or barley glucan per minute at pH 5.5 and 50 °C. OM, organic matter. DDGS, dried distiller grains with solubles. TDF, total dietary fiber.

## Data Availability

Not applicable.

## References

[1] Collins T., Gerday C., Feller G. (2005). Xylanases, xylanase families and extremophilic xylanases. FEMS Microbiol. Rev..

[2] McLoughlin R.F., Berthon B.S., Jensen M.E., Baines K.J., Wood L.G. (2017). Short-chain fatty acids, prebiotics, synbiotics, and systemic inflammation: A systematic review and meta-analysis. Am. J. Clin. Nutr..

[3] Prohászka L. (1986). Antibacterial Mechanism of Volatile Fatty Acids in the Intestinal Tract of Pigs agains *Escherichia coli*. J. Veter. Med. Ser. B.

[4] Passos A.A., Park I., Ferket P., von Heimendahl E., Kim S.W. (2015). Effect of dietary supplementation of xylanase on apparent ileal digestibility of nutrients, viscosity of digesta, and intestinal morphology of growing pigs fed corn and soybean meal-based diet. Anim. Nutr..

[5] Duarte M.E., Zhou F.X., Dutra W.M., Kim S.W. (2019). Dietary supplementation of xylanase and protease on growth performance, digesta viscosity, nutrient digestibility, immune and oxidative stress status, and gut health of newly weaned pigs. Anim. Nutr..

[6] Duarte M.E., Tyus J., Kim S.W. (2020). Synbiotic Effects of Enzyme and Probiotics on Intestinal Health and Growth of Newly Weaned Pigs Challenged with Enterotoxigenic F18+Escherichia coli. Front. Veter. Sci..

[7] Sakata T., Adachi M., Hashida M., Sato N., Kojima T. (1995). Effect of n-butyric acid on epithelial cell proliferation of pig colonic mucosa in short-term culture. DTW. Dtsch. Tierarztl. Wochenschr..

[8] Aragon C.C., Ruiz-Matute A.I., Corzo N., Monti R., Guisán J.M., Mateo C. (2018). Production of Xylo-oligosaccharides (XOS) by controlled hydrolysis of Xylan using immobilized Xylanase from Aspergillus niger with improved properties. Integr. Food Nutr. Metab..

[9] Qian E.W. (2014). Pretreatment and Saccharification of Lignocellulosic Biomass. Res. Approaches Sustain. Biomass Syst..

[10] Marquardt R.R., Li S. (2018). Antimicrobial resistance in livestock: Advances and alternatives to antibiotics. Anim. Front..

[11] Gibson G.R., Roberfroid M.B. (1995). Dietary Modulation of the Human Colonic Microbiota: Introducing the Concept of Prebiotics. J. Nutr..

[12] Wang T.-H., Lu S. (2013). Production of xylooligosaccharide from wheat bran by microwave assisted enzymatic hydrolysis. Food Chem..

[13] Jain I., Kumar V., Satyanarayana T. (2015). Xylooligosaccharides: An economical prebiotic from agroresidues and their health benefits. Indian J. Exp. Boil..

[14] Moeser A.J., Pohl C.S., Rajput M. (2017). Weaning stress and gastrointestinal barrier development: Implications for lifelong gut health in pigs. Anim. Nutr..

[15] Wijtten P.J.A., van der Meulen J., Verstegen M.W.A. (2011). Intestinal barrier function and absorption in pigs after weaning: A review. Br. J. Nutr..

[16] Jayaraman B., Nyachoti C.M. (2017). Husbandry practices and gut health outcomes in weaned piglets: A review. Anim. Nutr..

[17] Yang H., Xiong X., Wang X., Li T., Yin Y. (2016). Effects of weaning on intestinal crypt epithelial cells in piglets. Sci. Rep..

[18] Jia W., Slominski B.A., Bruce H.L., Blank G., Crow G., Jones O. (2009). Effects of diet type and enzyme addition on growth performance and gut health of broiler chickens during subclinical *Clostridium perfringens* challenge. Poult. Sci..

[19] Tiwari U.P., Fleming S.A., Rasheed M.S.A., Jha R., Dilger R.N. (2020). The role of oligosaccharides and polysaccharides of xylan and mannan in gut health of monogastric animals. J. Nutr. Sci..

[20] Zhong R., Ye Z.-H. (2015). Secondary Cell Walls: Biosynthesis, Patterned Deposition and Transcriptional Regulation. Plant. Cell Physiol..

[21] Bakker G., Dekker R., Jongbloed R., Jongbloed A. (1998). Non-starch polysaccharides in pig feeding. Veter. Q..

[22] Kim S.W., Knabe D.A., Hong K.J., Easter R.A. (2003). Use of carbohydrases in corn–soybean meal-based nursery diets1. J. Anim. Sci..

[23] Passos A.A., Andrade C., Phillips C.E., Coffey M.T., Kim S.W. (2015). Nutrient value of spray field forages fed to pigs and the use of feed enzymes to enhance nutrient digestibility. J. Anim. Sci..

[24] Tiwari U.P., Chen H., Kim S.W., Jha R. (2018). Supplemental effect of xylanase and mannanase on nutrient digestibility and gut health of nursery pigs studied using both in vivo and in vitro models. Anim. Feed. Sci. Technol..

[25] Choct M. (2015). Feed non-starch polysaccharides for monogastric animals: Classification and function. Anim. Prod. Sci..

[26] Annison G., Hughes R.J., Choct M. (1996). Effects of enzyme supplementation on the nutritive value of dehulled lupins. Br. Poult. Sci..

[27] Caprita R., Caprita A., Julean C. (2010). Biochemical aspects of non-starch polysaccharides. Anim. Sci. Bio-Technol..

[28] Choct M. (2006). Enzymes for the feed industry: Past, present and future. World’s Poult. Sci. J..

[29] Slominski B. (2011). Recent advances in research on enzymes for poultry diets. Poult. Sci..

[30] Ebringerová A., Hromádková Z., Heinze T. (2005). Hemicellulose. Advances in Polymer Science.

[31] Zhong R., Peña M.J., Zhou G.-K., Nairn C.J., Wood-Jones A., Richardson E.A., Iii W.H.M., Darvill A.G., York W.S., Ye Z.-H. (2005). Arabidopsis Fragile Fiber8, Which Encodes a Putative Glucuronyltransferase, Is Essential for Normal Secondary Wall Synthesis. Plant. Cell.

[32] Scheller H.V., Ulvskov P. (2010). Hemicelluloses. Annu. Rev. Plant. Biol..

[33] Köhnke T., Östlund Å., Brelid H. (2011). Adsorption of Arabinoxylan on Cellulosic Surfaces: Influence of Degree of Substitution and Substitution Pattern on Adsorption Characteristics. Biomacromolecules.

[34] Busse-Wicher M., Li A., Silveira R.L., Pereira C.S., Tryfona T., Gomes T.C.F., Skaf M.S., DuPree P. (2016). Evolution of Xylan Substitution Patterns in Gymnosperms and Angiosperms: Implications for Xylan Interaction with Cellulose. Plant. Physiol..

[35] Sedlmeyer F. (2011). Xylan as by-product of biorefineries: Characteristics and potential use for food applications. Food Hydrocoll..

[36] Peralta A.G., Venkatachalam S., Stone S.C., Pattathil S. (2017). Xylan epitope profiling: An enhanced approach to study organ development-dependent changes in xylan structure, biosynthesis, and deposition in plant cell walls. Biotechnol. Biofuels.

[37] Park Y.B., Cosgrove D.J. (2012). A Revised Architecture of Primary Cell Walls Based on Biomechanical Changes Induced by Substrate-Specific Endoglucanases. Plant. Physiol..

[38] Busse-Wicher M.N., Gomes T.C.F., Tryfona T., Nikolovski N., Stott K.M., Grantham N.J., Bolam D.N., Skaf M.S., DuPree P. (2014). The pattern of xylan acetylation suggests xylan may interact with cellulose microfibrils as a twofold helical screw in the secondary plant cell wall of Arabidopsis thaliana. Plant. J..

[39] Ebringerová A., Heinze T. (2000). Xylan and xylan derivatives—Biopolymers with valuable properties, 1: Naturally oc-curring xylans structures, isolation procedures and properties. Macromol. Rapid Commun..

[40] Hoebler C., Guillon F., Darcy-Vrillon B., Vaugelade P., Lahaye M., Worthington E., Duée P.H., Barry J.L. (2000). Supplementation of pig diet with algal fibre changes the chemical and physicochemical characteristics of diges-ta. J. Sci. Food Agric..

[41] Pettersson D., Pontoppi K. (2013). Soybean Meal and The Potential for Upgrading Its Feeding Value by Enzyme Sup-plementation. Soybean—Bio-Active Compounds.

[42] Piqué N., Gómez-Guillén M.D.C., Montero M.P. (2018). Xyloglucan, a Plant Polymer with Barrier Protective Properties over the Mucous Membranes: An Overview. Int. J. Mol. Sci..

[43] Verbruggen M.A., Spronk B.A., Schols H.A., Beldman G., Voragen A.G., Thomas J.R., Kamerling J.P., Vliegenthart J.F. (1998). Structures of enzymically derived oligosaccharides from sorghum glucuronoarabinoxylan. Carbohydr. Res..

[44] Knudsen K.E.B., Nørskov N.P., Bolvig A.K., Hedemann M.S., Laerke H.N. (2017). Dietary fibers and associated phytochemicals in cereals. Mol. Nutr. Food Res..

[45] Izydorczyk M.S., Biliaderis C.G. (1995). Cereal arabinoxylans: Advances in structure and physicochemical properties. Carbohydr. Polym..

[46] Viëtor R., Angelino S., Voragen A. (1992). Structural features of arabinoxylans from barley and malt cell wall material. J. Cereal Sci..

[47] Bengtsson S., Åman P. (1990). Isolation and chemical characterization of water-soluble arabinoxylans in rye grain. Carbohydr. Polym..

[48] Aspinall G., Carpenter R. (1984). Structural investigations on the non-starchy polysaccharides of oat bran. Carbohydr. Polym..

[49] Jaworski N.W., Lærke H.N., Knudsen K.E.B., Stein H.H. (2015). Carbohydrate composition and in vitro digestibility of dry matter and nonstarch polysaccharides in corn, sorghum, and wheat and coproducts from these grains. J. Anim. Sci..

[50] Lærke H.N., Arent S., Dalsgaard S., Knudsen K.E.B. (2015). Effect of xylanases on ileal viscosity, intestinal fiber modification, and apparent ileal fiber and nutrient digestibility of rye and wheat in growing pigs1,2. J. Anim. Sci..

[51] Pedersen M.B., Bunzel M., Schäfer J., Knudsen K.E.B., Sørensen J.F., Yu S., Lærke H.N. (2015). Ferulic Acid Dehydrodimer and Dehydrotrimer Profiles of Distiller’s Dried Grains with Solubles from Different Cereal Species. J. Agric. Food Chem..

[52] Tiwari U.P., Jha R. (2017). Nutrients, amino acid, fatty acid and non-starch polysaccharide profile and in vitro digestibility of macadamia nut cake in swine. Anim. Sci. J..

[53] Huntley N.F., Patience J.F. (2018). Xylose metabolism in the pig. PLoS ONE.

[54] Dhawan S., Kaur J. (2007). Microbial Mannanases: An Overview of Production and Applications. Crit. Rev. Biotechnol..

[55] O’Neill H.V.M., Smith J.A., Bedford M.R. (2014). Multicarbohydrase Enzymes for Non-ruminants. Asian-Australasian J. Anim. Sci..

[56] Teirlynck E., Haesebrouck F., Pasmans F., Dewulf J., Ducatelle R., van Immerseel F. (2009). The cereal type in feed influences *Salmonella enteritidis* colonization in broilers. Poult. Sci..

[57] Teirlynck E., Bjerrum L., Eeckhaut V., Huygebaert G., Pasmans F., Haesebrouck F., Dewulf J., Ducatelle R., van Immerseel F. (2009). The cereal type in feed influences gut wall morphology and intestinal immune cell infiltration in broiler chickens. Br. J. Nutr..

[58] Pettersson D., Graham H., Aman P. (1990). Enzyme supplementation of broiler chicken diets based on cereals with endosperm cell walls rich in arabinoxylans or mixed-linked β-glucans. Anim. Sci..

[59] Guenter W. (1993). Impact of Feed Enzymes on Nutrient Utilization of Ingredients in Growing Poultry. J. Appl. Poult. Res..

[60] Adeola O., Cowieson A.J. (2011). Boarded-Invited Review: Opportunities and challenges in using exogenous enzymes to improve nonruminant animal production. J. Anim. Sci..

[61] Kiarie E., Owusu-Asiedu A., Simmins P., Nyachoti C. (2010). Influence of phytase and carbohydrase enzymes on apparent ileal nutrient and standardized ileal amino acid digestibility in growing pigs fed wheat and barley-based diets. Livest. Sci..

[62] O’Neill H.V.M., Liu N., Wang J.P., Diallo A., Hill S. (2012). Effect of Xylanase on Performance and Apparent Metabolisable Energy in Starter Broilers Fed Diets Containing One Maize Variety Harvested in Different Regions of China. Asian-Australas. J. Anim. Sci..

[63] García M., Lázaro R., Latorre M., Gracia M., Mateos G. (2008). Influence of Enzyme Supplementation and Heat Processing of Barley on Digestive Traits and Productive Performance of Broilers. Poult. Sci..

[64] Yu X., Han J., Li H., Zhang Y., Feng J. (2018). The effect of enzymes on release of trace elements in feedstuffs based on in vitro digestion model for monogastric livestock. J. Anim. Sci. Biotechnol..

[65] Ferreira S.S., Passos C.P., Madureira P., Vilanova M., Coimbra M.A. (2015). Structure–function relationships of immunostimulatory polysaccharides: A review. Carbohydr. Polym..

[66] Shastak Y., Ader P., Feuerstein D., Ruehle R., Matuschek M. (2015). ß-Mannan and mannanase in poultry nutrition. World’s Poult. Sci. J..

[67] De Servi B., Ranzini F., Piqué N. (2017). Protective barrier properties of Rhinosectan^®^ spray (containing xyloglucan) on an organotypic 3D airway tissue model (MucilAir): Results of an in vitro study. Allergy Asthma Clin. Immunol..

[68] De Servi B., Ranzini F., Piqué N. (2016). Effect of Utipro^®^ (containing gelatin-xyloglucan) against *Escherichia coli* invasion of intestinal epithelial cells: Results of anin vitrostudy. Futur. Microbiol..

[69] Fraile B., Alcover J., Royuela M., Rodríguez D., Chaves C., Palacios R., Piqué N. (2017). Xyloglucan, hibiscus and propolis for the prevention of urinary tract infections: Results ofin vitrostudies. Futur. Microbiol..

[70] Mishra A., Malhotra A.V. (2010). ChemInform Abstract: Tamarind Xyloglucan: A Polysaccharide with Versatile Application Potential. J. Mater. Chem..

[71] Tauzin A.S., Kwiatkowski K.J., Orlovsky N.I., Smith C.J., Creagh A.L., Haynes C.A., Wawrzak Z., Brumer H., Koropatkin N.M. (2016). Molecular Dissection of Xyloglucan Recognition in a Prominent Human Gut Symbiont. mBio.

[72] Martinez-Fleites C., Guerreiro C.I.P.D., Baumann M.J., Taylor E.J., Prates J.A.M., Ferreira L.M.A., Fontes C.M.G.A., Brumer H., Davies G.J. (2006). Crystal Structures of Clostridium thermocellum Xyloglucanase, XGH74A, Reveal the Structural Basis for Xyloglucan Recognition and Degradation. J. Biol. Chem..

[73] Cantu-Jungles T.M., Nascimento G.E.D., Zhang X., Iacomini M., Cordeiro L.M., Hamaker B.R. (2019). Soluble xyloglucan generates bigger bacterial community shifts than pectic polymers during in vitro fecal fermentation. Carbohydr. Polym..

[74] Ward N.E. (2014). Choosing enzyme solution depends on many factors. Feedstuffs.

[75] Knudsen K.E.B. (2014). Fiber and nonstarch polysaccharide content and variation in common crops used in broiler diets. Poult. Sci..

[76] Knudsen K.E.B. (1997). Carbohydrate and lignin contents of plant materials used in animal feeding. Anim. Feed. Sci. Technol..

[77] Irish G., Balnave D. (1993). Non-starch polysaccharides and broiler performance on diets containing soyabean meal as the sole protein concentrate. Aust. J. Agric. Res..

[78] Englyst H.N., Bingham S.A., Runswick S.A., Collinson E., Cummings J.H. (1989). Dietary fibre (non-starch polysaccharides) in cereal products. J. Hum. Nutr. Diet..

[79] Bautil A., Verspreet J., Buyse J., Goos P., Bedford M., Courtin C. (2020). Arabinoxylan-oligosaccharides kick-start arabinoxylan digestion in the aging broiler. Poult. Sci..

[80] Courtin C.M., Swennen K., Broekaert W.F., Swennen Q., Buyse J., Decuypere E., Michiels C.W., de Ketelaere B., Delcour J.A. (2008). Effects of dietary inclusion of xylooligo- saccharides, arabinoxylooligosaccha- rides and soluble arabinoxylan on the microbial composition of caecal contents of chickens. J. Sci. Food Agric..

[81] Schutte J.B. (1990). Nutritional Implications and Metabolizable Energy Value of D-Xylose and L-Arabinose in Chicks. Poult. Sci..

[82] Chen H., Wang W., de Groote J., Possemiers S., Chen D., de Smet S., Michiels J. (2015). Arabinoxylan in Wheat Is More Responsible Than Cellulose for Promoting Intestinal Barrier Function in Weaned Male Piglets. J. Nutr..

[83] Chen H., Zhang S., Kim S.W. (2020). Effects of supplemental xylanase on health of the small intestine in nursery pigs fed diets with corn distillers’ dried grains with solubles. J. Anim. Sci..

[84] Jørgensen H., Zhao X.-Q., Eggum B.O. (1996). The influence of dietary fibre and environmental temoperature on the development of the gastrointestinal tract, digestibility, degree of fermentation in the hind-gut and energy metabolism in pigs. Br. J. Nutr..

[85] Sun H., Cozannet P., Ma R., Zhang L., Huang Y.-K., Preynat A., Sun L.-H. (2020). Effect of concentration of arabinoxylans and a carbohydrase mixture on energy, amino acids and nutrients total tract and ileal digestibility in wheat and wheat by-product-based diet for pigs. Anim. Feed. Sci. Technol..

[86] Akpinar O., Erdogan K., Bostanci S. (2009). Production of xylooligosaccharides by controlled acid hydrolysis of lignocellulosic materials. Carbohydr. Res..

[87] Brienzo M., Carvalho W., Milagres A.M.F. (2010). Xylooligosaccharides Production from Alkali-Pretreated Sugarcane Bagasse Using Xylanases from *Thermoascus aurantiacus*. Appl. Biochem. Biotechnol..

[88] Akpinar O., Ak O., Kavas A., Bakir A.U., Yilmaz L. (2007). Enzymatic Production of Xylooligosaccharides from Cotton Stalks. J. Agric. Food Chem..

[89] Teng C., Yan Q., Jiang Z., Fan G., Shi B. (2010). Production of xylooligosaccharides from the steam explosion liquor of corncobs coupled with enzymatic hydrolysis using a thermostable xylanase. Bioresour. Technol..

[90] Nabarlatz D.A., Farriol X., Montané D. (2005). Autohydrolysis of Almond Shells for the Production of Xylo-oligosaccharides: Product Characteristics and Reaction Kinetics. Ind. Eng. Chem. Res..

[91] Yang R., Xu S., Wang Z., Yang W. (2005). Aqueous extraction of corncob xylan and production of xylooligosaccharides. LWT.

[92] Garrote G., Domínguez H., Parajó J.C. (2004). Production of Substituted Oligosaccharides by Hydrolytic Processing of Barley Husks. Ind. Eng. Chem. Res..

[93] Kabel M. (2002). Hydrothermally treated xylan rich by-products yield different classes of xylo-oligosaccharides. Carbohydr. Polym..

[94] Aachary A.A., Prapulla S.G. (2009). Value addition to corncob: Production and characterization of xylooligosaccharides from alkali pretreated lignin-saccharide complex using Aspergillus oryzae MTCC 5154. Bioresour. Technol..

[95] Aachary A.A., Prapulla S.G. (2010). Xylooligosaccharides (XOS) as an Emerging Prebiotic: Microbial Synthesis, Utilization, Structural Characterization, Bioactive Properties, and Applications. Compr. Rev. Food Sci. Food Saf..

[96] Swennen K., Courtin C.M., van der Bruggen B., Vandecasteele C., Delcour J.A. (2005). Ultrafiltration and ethanol precipitation for isolation of arabinoxylooligosaccharides with different structures. Carbohydr. Polym..

[97] Vázquez M., Garrote G., Alonso J., Domínguez H., Parajó J. (2005). Refining of autohydrolysis liquors for manufacturing xylooligosaccharides: Evaluation of operational strategies. Bioresour. Technol..

[98] Vegas R., Alonso J.L., Domínguez H., Parajó J.C. (2004). Processing of Rice Husk Autohydrolysis Liquors for Obtaining Food Ingredients. J. Agric. Food Chem..

[99] Vegas R., Alonso J.L., Domínguez H., Parajó J.C. (2005). Manufacture and Refining of Oligosaccharides from Industrial Solid Wastes. Ind. Eng. Chem. Res..

[100] Nabarlatz D., Farriol X., Montané D. (2004). Kinetic Modeling of the Autohydrolysis of Lignocellulosic Biomass for the Production of Hemicellulose-Derived Oligosaccharides. Ind. Eng. Chem. Res..

[101] Carvalheiro F., Esteves M.P., Parajó J.C., Pereira H., Gírio F.M. (2004). Production of oligosaccharides by autohydrolysis of brewery’s spent grain. Bioresour. Technol..

[102] Carvalheiro F., Garrote G., Parajó J.C., Pereira H., Gírio F.M. (2008). Kinetic Modeling of Brewery’s Spent Grain Autohydrolysis. Biotechnol. Prog..

[103] Kim Y., Hendrickson R., Mosier A.N., Ladisch M.R. (2005). Plug-Flow Reactor for Continuous Hydrolysis of Glucans and Xylans from Pretreated Corn Fiber. Energy Fuels.

[104] Palm M., Zacchi G. (2003). Extraction of Hemicellulosic Oligosaccharides from Spruce Using Microwave Oven or Steam Treatment. Biomacromolecules.

[105] Qing Q., Li H., Kumar R., Wyman C.E. (2013). Xylooligosaccharides Production, Quantification, and Characterization in Context of Lignocellulosic Biomass Pretreatment. Aqueous Pretreatment of Plant Biomass for Biological and Chemical Conversion to Fuels and Chemicals.

[106] Ai Z., Jiang Z., Li L., Deng W., Kusakabe I., Li H. (2005). Immobilization of *Streptomyces olivaceoviridis* E-86 xylanase on Eudragit S-100 for xylo-oligosaccharide production. Process. Biochem..

[107] Yoon K.Y., Woodams E.E., Hang Y.D. (2006). Enzymatic production of pentoses from the hemicellulose fraction of corn residues. LWT Food Sci. Technol..

[108] Zilliox C., Debeire P. (1998). Hydrolysis of wheat straw by a thermostable endoxylanase: Adsorption and kinetic studies. Enzym. Microb. Technol..

[109] Chen C., Chen J.-L., Lin T.-Y. (1997). Purification and characterization of a xylanase from *Trichoderma longibrachiatum* for xylooligosaccharide production. Enzym. Microb. Technol..

[110] Freixo M., de Pinho M.N. (2002). Enzymatic hydrolysis of beechwood xylan in a membrane reactor. Desalination.

[111] Nishimura T., Ishihara M., Ishii T., Kato A. (1998). Structure of neutral branched xylooligosaccharides produced by xylanase from in situ reduced hardwood xylan. Carbohydr. Res..

[112] Okazaki M., Fujikawa S., Matsumoto N. (1990). Effect of Xylooligosaccharide on the Growth of Bifidobacteria. Bifid Microflora.

[113] Celi P., Verlhac V., Calvo E.P., Schmeisser J., Kluenter A.-M. (2019). Biomarkers of gastrointestinal functionality in animal nutrition and health. Anim. Feed. Sci. Technol..

[114] Montagne L., Pluske J., Hampson D. (2003). A review of interactions between dietary fibre and the intestinal mucosa, and their consequences on digestive health in young non-ruminant animals. Anim. Feed. Sci. Technol..

[115] Jha R., Fouhse J.M., Tiwari U.P., Li L., Willing B.P. (2019). Dietary Fiber and Intestinal Health of Monogastric Animals. Front. Vet. Sci..

[116] Seo Y.S., Shah V.H. (2012). The role of gut-liver axis in the pathogenesis of liver cirrhosis and portal hypertension. Clin. Mol. Hepatol..

[117] Liu Z., Li N., Neu J. (2007). Tight junctions, leaky intestines, and pediatric diseases. Acta Paediatr..

[118] Amaretti A., di Nunzio M., Pompei A., Raimondi S., Rossi M., Bordoni A. (2013). Antioxidant properties of potentially probiotic bacteria: In vitro and in vivo activities. Appl. Microbiol. Biotechnol..

[119] Smiricky-Tjardes M.R., Flickinger E.A., Grieshop C.M., Bauer L.L., Murphy M.R., Fahey G.C. (2003). In vitro fermentation characteristics of selected oligosaccharides by swine fecal microflora1. J. Anim. Sci..

[120] Imoto S., Namioka S. (1978). VFA Production in the Pig Large Intestine. J. Anim. Sci..

[121] Finegold S.M., Li Z., Summanen P.H., Downes J., Thames G., Corbett K., Dowd S., Krak M., Heber D. (2014). Xylooligosaccharide increases bifidobacteria but not lactobacilli in human gut microbiota. Food Funct..

[122] Sokol H., Pigneur B., Watterlot L., Lakhdari O., Bermúdez-Humarán L.G., Gratadoux J.-J., Blugeon S., Bridonneau C., Furet J.-P., Corthier G. (2008). Faecalibacterium prausnitzii is an anti-inflammatory commensal bacterium identified by gut microbiota analysis of Crohn disease patients. Proc. Natl. Acad. Sci. USA.

[123] Collado M.C., Derrien M., Isolauri E., de Vos W.M., Salminen S. (2007). Intestinal Integrity and *Akkermansia muciniphila*, a Mucin-Degrading Member of the Intestinal Microbiota Present in Infants, Adults, and the Elderly. Appl. Environ. Microbiol..

[124] Jaskari J., Kontula P., Siitonen A., Jousimies-Somer H., Mattila-Sandholm T., Poutanen K. (1998). Oat β-glucan and xylan hydrolysates as selective substrates for *Bifidobacterium* and *Lactobacillus strains*. Appl. Microbiol. Biotechnol..

[125] Van Laere K.M.J., Hartemink R., Bosveld M., Schols H.A., Voragen A.G.J. (2000). Fermentation of Plant Cell Wall Derived Polysaccharides and Their Corresponding Oligosaccharides by Intestinal Bacteria. J. Agric. Food Chem..

[126] Pan J., Yin J., Zhang K., Xie P., Ding H., Huang X., Blachier F., Kong X. (2019). Dietary xylo-oligosaccharide supplementation alters gut microbial composition and activity in pigs according to age and dose. AMB Express.

[127] Azad M.A.K., Sarker M., Li T., Yin J. (2018). Probiotic Species in the Modulation of Gut Microbiota: An Overview. BioMed Res. Int..

[128] Liu J., Cao S.C., Xie Y.N., Zhang H.F. (2018). Effect of probiotics and xylo-oligosaccharide supplementation on nutrient digestibility, intestinal health and noxious gas emission in weanling pigs. Asian-Australas. J. Anim. Sci..

[129] Yin J., Li F., Kong X., Wen C., Guo Q., Zhang L., Wang W., Duan Y., Li T., Tan Z. (2019). Dietary xylo-oligosaccharide improves intestinal functions in weaned piglets. Food Funct..

[130] Madhukumar M.S., Muralikrishna G. (2011). Fermentation of xylo-oligosaccharides obtained from wheat bran and Bengal gram husk by lactic acid bacteria and bifidobacteria. J. Food Sci. Technol..

[131] Crittenden R., Karppinen S., Ojanen S., Tenkanen M., Fagerström R., Mättö J., Saarela M., Mattila-Sandholm T., Poutanen K. (2002). In vitrofermentation of cereal dietary fibre carbohydrates by probiotic and intestinal bacteria. J. Sci. Food Agric..

[132] Swennen K., Courtin C.M., Delcour J.A. (2006). Non-digestible Oligosaccharides with Prebiotic Properties. Crit. Rev. Food Sci. Nutr..

[133] Gibson G., Wang X. (1994). Regulatory effects of bifidobacteria on the growth of other colonic bacteria. J. Appl. Bacteriol..

[134] Lin S.-H., Chou L.-M., Chien Y.-W., Chang J.-S., Lin C.-I. (2016). Prebiotic Effects of Xylooligosaccharides on the Improvement of Microbiota Balance in Human Subjects. Gastroenterol. Res. Pr..

[135] Uzal F.A., McClane B.A., Cheung J.K., Theoret J., Garcia J.P., Moore R.J., Rood J.I. (2015). Animal models to study the pathogenesis of human and animal *Clostridium perfringens* infections. Veter. Microbiol..

[136] Hsu C.-K., Liao J.-W., Chung Y.-C., Hsieh C.-P., Chan Y.-C. (2004). Xylooligosaccharides and Fructooligosaccharides Affect the Intestinal Microbiota and Precancerous Colonic Lesion Development in Rats. J. Nutr..

[137] Howard M.D., Gordon D.T., Garleb K.A., Kerley M.S. (1995). Dietary fructooligosaccharide, xylooligosaccharide and gum arabic have variable effects on cecal and colonic microbiota and epithelial cell proliferation in mice and rats. J. Nutr..

[138] Tomomatsu H. (1994). Health effects of oligosaccharides. Food Technol..

[139] Samanta A.K., Jayapal N., Jayaram C., Roy S., Kolte A.P., Senani S., Sridhar M. (2015). Xylooligosaccharides as prebiotics from agricultural by-products: Production and applications. Bioact. Carbohydr. Diet. Fibre.

[140] Danicke S., Vahjen W., Simon O., Jeroch H. (1999). Effects of dietary fat type and xylanase supplementation to rye-based broiler diets on selected bacterial groups adhering to the intestinal epithelium. on transit time of feed, and on nutrient digestibility. Poult. Sci..

[141] Suo H.-Q., Lu L., Xu G.-H., Xiao L., Chen X.-G., Xia R.-R., Zhang L.-Y., Luo X.-G. (2015). Effectiveness of dietary xylo-oligosaccharides for broilers fed a conventional corn-soybean meal diet. J. Integr. Agric..

[142] De Maesschalck C., Eeckhaut V., Maertens L., de Lange L., Marchal L., Nezer C., de Baere S., Croubels S., Daube G., Dewulf J. (2015). Effects of Xylo-Oligosaccharides on Broiler Chicken Performance and Microbiota. Appl. Environ. Microbiol..

[143] Sun Z., Lv W., Yu R., Li J., Liu H., Sun W., Wang Z., Li J., Zhe S., Qin Y. (2013). Effect of a straw-derived xylooligo-saccharide on broiler growth performance, endocrine metabolism, and immune response. Can. J. Vet. Res..

[144] Samanta A.K., Kolte A.P., Elangovan A.V., Dhali A., Senani S., Sridhar M., Jayapal N. (2017). Effects of corn husks derived xylooligosaccharides on performance of broiler chicken. Indian J. Anim. Sci..

[145] Craig A.D., Khattak F., Hastie P., Bedford M.R., Olukosi O.A. (2020). Xylanase and xylo- oligosaccharide prebiotic improve the growth performance and concentration of potentially prebiotic oligosaccharides in the ileum of broiler chickens. Br. Poult. Sci..

[146] Craig A.D., Khattak F., Hastie P., Bedford M.R., Olukosi O.A. (2020). The similarity of the effect of carbohydrase or prebiotic supplementation in broilers aged 21 days, fed mixed cereal diets and challenged with coccidiosis infection. PLoS ONE.

[147] Li D., Ding X., Zhang K., Bai S., Wang J., Zeng Q., Su Z., Kang L. (2017). Effects of dietary xylooligosaccharides on the performance, egg quality, nutrient digestibility and plasma parameters of laying hens. Anim. Feed. Sci. Technol..

[148] Van Der-Klis J.D., van Voorst A., van Cruyningen C. (1993). Effect of a soluble polysaccharide (carboxy methyl cellulose) on the physico-chemical conditions in the gastrointestinal tract of broilers. Br. Poult. Sci..

[149] Aronsson A., Karlsson E.N. (2017). Structural Considerations on the Use of Endo-Xylanases for the Production of prebiotic Xylooligosaccharides from Biomass. Curr. Protein Pept. Sci..

[150] Wong K.K., Tan L.U., Saddler J.N. (1988). Multiplicity of beta-1,4-xylanase in microorganisms: Functions and applica-tions. Microbiol. Rev..

[151] Henrissat B., Claeyssens M., Tomme P., Lemesle L., Mornon J.-P. (1989). Cellulase families revealed by hydrophobic cluster analysi. Gene.

[152] Henrissat B., Coutinho P.M. (2001). Classification of glycoside hydrolases and glycosyltransferases from hyperthermophiles. Cellulases.

[153] Bourne Y., Henrissat B. (2001). Glycoside hydrolases and glycosyltransferases: Families and functional modules. Curr. Opin. Struct. Biol..

[154] Singh S., Madlala A.M., Prior B.A. (2003). Thermomyces lanuginosus: Properties of strains and their hemicellulases. FEMS Microbiol. Rev..

[155] Subramaniyan S., Prema P. (2002). Biotechnology of Microbial Xylanases: Enzymology, Molecular Biology, and Application. Crit. Rev. Biotechnol..

[156] Sunna A., Antranikian G. (1997). Xylanolytic Enzymes from Fungi and Bacteria. Crit. Rev. Biotechnol..

[157] Jeffries T.W. (1996). Biochemistry and genetics of microbial xylanases. Curr. Opin. Biotechnol..

[158] Aragon C.C., Mateo C., Ruiz-Matute A.I., Corzo N., Fernandez-Lorente G., Sevillano L., Diaz M., Monti R., Santamaría R.I., Guisán J.M. (2013). Production of xylo-oligosaccharides by immobilized-stabilized derivatives of endo-xylanase from *Streptomyces halstedii*. Process. Biochem..

[159] Yan Q., Hao S., Jiang Z., Zhai Q., Chen W. (2009). Properties of a xylanase from *Streptomyces matensis* being suitable for xylooligosaccharides production. J. Mol. Catal. B: Enzym..

[160] Abou-Hachem M., Olsson F., Karlsson E.N. (2003). Probing the stability of the modular family 10 xylanase from *Rhodothermus marinus*. Extrem..

[161] Beaugrand J., Chambat G., Wong V.W., Goubet F., Rémond C., Paës G., Benamrouche S., Debeire P., O’Donohue M., Chabbert B. (2004). Impact and efficiency of GH10 and GH11 thermostable endoxylanases on wheat bran and alkali-extractable arabinoxylans. Carbohydr. Res..

[162] Biely P., Vršanská M., Tenkanen M., Kluepfel D. (1997). Endo-β-1,4-xylanase families: Differences in catalytic properties. J. Biotechnol..

[163] John F.J.S., González J.M., Pozharski E. (2010). Consolidation of glycosyl hydrolase family 30: A dual domain 4/7 hydrolase family consisting of two structurally distinct groups. FEBS Lett..

[164] Paës G., Berrin J.-G., Beaugrand J. (2012). GH11 xylanases: Structure/function/properties relationships and applications. Biotechnol. Adv..

[165] Luo H., Yang J., Li J., Shi P., Huang H., Bai Y., Fan Y., Yao B. (2010). Molecular cloning and characterization of the novel acidic xylanase XYLD from Bispora sp. MEY-1 that is homologous to family 30 glycosyl hydrolases. Appl. Microbiol. Biotechnol..

[166] John F.J.S., Godwin D.K., Preston J.F., Pozharski E., Hurlbert J.C. (2009). Crystallization and crystallographic analysis of *Bacillus subtilisxylanase* C. Acta Crystallogr. Sect. F Struct. Biol. Cryst. Commun..

[167] John F.J.S., Hurlbert J.C., Rice J.D., Preston J.F., Pozharski E. (2011). Ligand Bound Structures of a Glycosyl Hydrolase Family 30 Glucuronoxylan Xylanohydrolase. J. Mol. Biol..

[168] Larson S.B., Day J., de la Rosa A.P.B., Keen N.T., McPherson A. (2003). First Crystallographic Structure of a Xylanase from Glycoside Hydrolase Family 5: Implications for Catalysis. Biochemistry.

[169] Urbanikova L., Vršanská M., Krogh K.B.R.M., Hoff T., Biely P. (2011). Structural basis for substrate recognition by *Erwinia chrysanthemi* GH30 glucuronoxylanase. FEBS J..

[170] Sainz-Polo M.A., Valenzuela S.V., González B., Pastor F.I.J., Sanz-Aparicio J. (2014). Structural Analysis of Glucuronoxylan-specific Xyn30D and Its Attached CBM35 Domain Gives Insights into the Role of Modularity in Specificity. J. Biol. Chem..

[171] John F.J.S., Dietrich D., Crooks C., Pozharski E., González J.M., Bales E., Smith K., Hurlbert J.C. (2014). A novel member of glycoside hydrolase family 30 subfamily 8 with altered substrate specificity. Acta Crystallogr. Sect. D Biol. Crystallogr..

[172] Torres-Pitarch A., Manzanilla E., Gardiner G., O’Doherty J., Lawlor P. (2019). Systematic review and meta-analysis of the effect of feed enzymes on growth and nutrient digestibility in grow-finisher pigs: Effect of enzyme type and cereal source. Anim. Feed. Sci. Technol..

[173] Bedford M.R. (2018). The evolution and application of enzymes in the animal feed industry: The role of data interpretation. Br. Poult. Sci..

[174] Diebold G., Mosenthin R., Piepho H.-P., Sauer W.C. (2004). Effect of supplementation of xylanase and phospholipase to a wheat-based diet for weanling pigs on nutrient digestibility and concentrations of microbial metabolites in ileal digesta and feces1. J. Anim. Sci..

[175] Yin Y.-L., McEvoy J., Schulze H., Hennig U., Souffrant W.-B., McCracken K. (2000). Apparent digestibility (ileal and overall) of nutrients and endogenous nitrogen losses in growing pigs fed wheat (var. Soissons) or its by-products without or with xylanase supplementation. Livest. Prod. Sci..

[176] Jørgensen H., Serena A., Hedemann M.S., Knudsen K.E.B. (2007). The fermentative capacity of growing pigs and adult sows fed diets with contrasting type and level of dietary fibre. Livest. Sci..

[177] Urriola P.E., Stein H.H. (2012). Comparative digestibility of energy and nutrients in fibrous feed ingredients fed to Meishan and Yorkshire pigs. J. Anim. Sci..

[178] Kerkaert H.R., de Rouchey J.M., Dritz S.S., Goodband R.D., Tokach M.D., Woodworth J.C., Calderone-Cartagena H., Gonçalves M. (2019). Determining the Effects of Increasing Levels of Xylanase in Nutrient Adequate Diets on Growth Performance, Carcass Characteristics of Growing-Finishing Pigs. Kans. Agric. Exp. Stn. Res. Rep..

[179] Debyser W., Peumans W., van Damme E., Delcour J. (1999). *Triticum aestivum* Xylanase Inhibitor (TAXI), a New Class of Enzyme Inhibitor Affecting Breadmaking Performance. J. Cereal Sci..

[180] Mclauchlan W.R., Garcia-Conesa M.T., Williamson G., Roza M., Ravestein P., Maat J. (1999). A novel class of protein from wheat which inhibits xylanases1. Biochem. J..

[181] Goesaert H., Debyser W., Gebruers K., Proost P., van Damme J., Delcour J.A. (2001). Purification and Partial Characterization of an Endoxylanase Inhibitor from Barley. Cereal Chem. J..

[182] Goesaert H., Gebruers K., Courtin C., Proost P., van Damme J., Delcour J. (2002). A Family of ‘TAXI’-like Endoxylanase Inhibitors in Rye. J. Cereal Sci..

[183] Goesaert H., Elliott G., Kroon P.A., Gebruers K., Courtin C.M., Robben J., Delcour J.A., Juge N. (2004). Occurrence of proteinaceous endoxylanase inhibitors in cereals. Biochim. Biophys. Acta Proteins Proteom..

[184] Fierens E., Rombouts S., Gebruers K., Goesaert H., Brijs K., Beaugrand J., Volckaert G., van Campenhout S., Proost P., Courtin C.M. (2007). TLXI, a novel type of xylanase inhibitor from wheat (*Triticum aestivum*) belonging to the thaumatin family. Biochem. J..

[185] Flatman R., Mclauchlan R.W., Juge N., Furniss C., Berrin J.-G., Hughes R.K., Manzanares P., Ladbury J.E., O’Brien R., Williamson G. (2002). Interactions defining the specificity between fungal xylanases and the xylanase-inhibiting protein XIP-I from wheat. Biochem. J..

[186] Gebruers K., Dornez E., Bedõ Z., Rakszegi M., Courtin C.M., Delcour J.A., Rakszegi M. (2010). Variability in Xylanase and Xylanase Inhibition Activities in Different Cereals in the HEALTHGRAIN Diversity Screen and Contribution of Environment and Genotype to This Variability in Common Wheat. J. Agric. Food Chem..

[187] Sancho A.I., Faulds C.B., Svensson B., Bartolomé B., Williamson G., Juge N. (2003). Cross-inhibitory activity of cereal protein inhibitors against α-amylases and xylanases. Biochim. Biophys. Acta Proteins Proteom..

[188] Juge N., Payan F., Williamson G. (2004). XIP-I, a xylanase inhibitor protein from wheat: A novel protein function. Biochim. Biophys. Acta Proteins Proteom..

[189] Elliott G., Mc Lauchlan W.R., Williamson G., Kroon P. (2003). A Wheat Xylanase Inhibitor Protein (XIP-I) Accumulates in the Grain and has Homologues in Other Cereals. J. Cereal Sci..

[190] Goesaert H., Gebruers K., Brijs K., Courtin C., Delcour J. (2003). XIP-type endoxylanase inhibitors in different cereals. J. Cereal Sci..

[191] Goesaert H., Gebruers K., Courtin C.M., Delcour J.A. (2005). Purification and characterization of a XIP-type endoxylanase inhibitor from Rice (*Oryza sativa*). J. Enzym. Inhib. Med. Chem..

[192] Durand A., Hughes R., Roussel A., Flatman R., Henrissat B., Juge N. (2005). Emergence of a subfamily of xylanase inhibitors within glycoside hydrolase family 18. FEBS J..

[193] Beaugrand J., Gebruers K., Ververken C., Fierens E., Croes E., Goddeeris B., Courtin C.M., Delcour J.A. (2006). Antibodies against wheat xylanase inhibitors as tools for the selective identification of their homologues in other cereals. J. Cereal Sci..

[194] Mokrane H., Gebruers K., Beaugrand J., Proost P., Nadjemi B., Belhanèche-Bensemra N., Courtin C.M., Delcour J.A. (2009). Algerian Pearl Millet (*Pennisetum glaucum* L.) Contains XIP but Not TAXI and TLXI Type Xylanase Inhibitors. J. Agric. Food Chem..

[195] Croes E., Gebruers K., Robben J., Noben J.-P., Samyn B., Debyser G., van Beeumen J., Delcour J.A., Courtin C.M. (2008). Variability of polymorphic families of three types of xylanase inhibitors in the wheat grain proteome. Proteomics.

[196] Croes E., Gebruers K., Carpentier S., Swennen R., Robben J., Laukens K., Witters E., Delcour J.A., Courtin C.M. (2009). A quantitative portrait of three xylanase inhibiting protein families in different wheat cultivars using 2D-DIGE and multivariate statistical tools. J. Proteom..

[197] Bonnin E., Daviet S., Gebruers K., Delcour J.A., Goldson A., Juge N., Saulnier L. (2005). Variation in the levels of the different xylanase inhibitors in grain and flour of 20 French wheat cultivars. J. Cereal Sci..

[198] Liu W.-C., Kim I.-H. (2017). Effects of dietary xylanase supplementation on performance and functional digestive parameters in broilers fed wheat-based diets. Poult. Sci..

[199] Lee S., Apajalahti J., Vienola K., González-Ortiz G., Fontes C., Bedford M. (2017). Age and dietary xylanase supplementation affects ileal sugar residues and short chain fatty acid concentration in the ileum and caecum of broiler chickens. Anim. Feed. Sci. Technol..

[200] González-Ortiz G., Olukosi O., Bedford M.R. (2016). Evaluation of the effect of different wheats and xylanase supplementation on performance, nutrient and energy utilisation in broiler chicks. Anim. Nutr..

[201] Olukosi O., Bedford M. (2019). Comparative effects of wheat varieties and xylanase supplementation on growth performance, nutrient utilization, net energy, and whole-body energy and nutrient partitioning in broilers at different ages. Poult. Sci..

[202] McCafferty K., Bedford M., Kerr B., Dozier W. (2019). Effects of cereal grain source and supplemental xylanase concentrations on broiler growth performance and cecal volatile fatty acid concentrations from 1 to 40 d of age. Poult. Sci..

[203] Masey-O’Neill H., Singh M., Cowieson A. (2014). Effects of exogenous xylanase on performance, nutrient digestibility, volatile fatty acid production and digestive tract thermal profiles of broilers fed on wheat- or maize-based diet. Br. Poult. Sci..

[204] Walk C., Poernama F. (2019). Evaluation of Phytase, Xylanase, and Protease in Reduced Nutrient Diets Fed to Broilers. J. Appl. Poult. Res..

[205] Liu D., Guo S., Guo Y. (2012). Xylanase supplementation to a wheat-based diet alleviated the intestinal mucosal barrier impairment of broiler chickens challenged by *Clostridium perfringens*. Avian Pathol..

[206] Singh A.V., O’Neill H.M., Ghosh T., Bedford M., Haldar S. (2012). Effects of xylanase supplementation on performance, total volatile fatty acids and selected bacterial population in caeca, metabolic indices and peptide YY concentrations in serum of broiler chickens fed energy restricted maize–soybean-based diets. Anim. Feed. Sci. Technol..

[207] Cowieson A., O’Neill H.M., O’Neill H.M. (2013). Effects of exogenous xylanase on performance, nutrient digestibility and caecal thermal profiles of broilers given wheat-based diets. Br. Poult. Sci..

[208] Tsai T., Dove C., Cline P., Owusu-Asiedu A., Walsh M., Azain M. (2017). The effect of adding xylanase or β-glucanase to diets with corn distillers dried grains with solubles (CDDGS) on growth performance and nutrient digestibility in nursery pigs. Livest. Sci..

[209] Taylor A.E., Bedford M.R., Miller H.M. (2018). The effects of xylanase on grower pig performance, concentrations of volatile fatty acids and peptide YY in portal and peripheral blood. Animals.

[210] Lee K.Y., Balasubramanian B., Kim J.K., Kim I.H. (2018). Dietary inclusion of xylanase improves growth performance, apparent total tract nutrient digestibility, apparent ileal digestibility of nutrients and amino acids and alters gut microbiota in growing pigs. Anim. Feed. Sci. Technol..

[211] Moran K., de Lange C.F.M., Ferket P., Fellner V., Wilcock P., van Heugten E. (2016). Enzyme supplementation to improve the nutritional value of fibrous feed ingredients in swine diets fed in dry or liquid form1. J. Anim. Sci..

[212] Petry A.L., O’Neill H.V.M., Patience J.F. (2019). Xylanase, and the role of digestibility and hindgut fermentation in pigs on energetic differences among high and low energy corn samples1. J. Anim. Sci..

